# Range‐extending fish become competitive dominants under ocean warming but not heatwaves or acidification

**DOI:** 10.1002/ecy.70226

**Published:** 2026-02-11

**Authors:** Angus Mitchell, Ericka O. C. Coni, Sean D. Connell, David J. Booth, Ben P. Harvey, Sylvain Agostini, Timothy Ravasi, Ivan Nagelkerken

**Affiliations:** ^1^ Southern Seas Ecology Laboratories, School of Biological Sciences The University of Adelaide Adelaide South Australia Australia; ^2^ School of the Life Sciences University of Technology Sydney Ultimo New South Wales Australia; ^3^ Shimoda Marine Research Center University of Tsukuba Shimoda Shizuoka Japan; ^4^ Labex ICONA International CO_2_ Natural Analogues Network Japan; ^5^ Marine Climate Change Unit Okinawa Institute of Science and Technology (OIST) Onna‐son Okinawa Japan; ^6^ College of Science and Engineering James Cook University Townsville Queensland Australia; ^7^ Present address: Entropie, IRD, IFREMER, Univ La Réunion, Univ. Nouvelle‐Calédonie Nouméa New Caledonia

**Keywords:** climate change, marine heatwaves, ocean acidification, ocean warming, range shifts, reef fishes, species interactions, species redistributions

## Abstract

Ocean warming is driving species range extensions into cooler regions. The direct physiological influence of warming on species performance can accelerate such extensions into novel ecosystems; however, indirect effects of invader–resident interactions in cooler regions may counter these positive effects. Here, we examined the foraging performance and densities of competing warm‐water and cool‐water fishes across a latitudinal temperature gradient spanning 1500 km from tropical to temperate reefs subjected to rapid ocean warming in the southern hemisphere, and across natural analogs of temperate, tropicalized, and acidified reef localities in the northern hemisphere, and during a severe marine heatwave at a temperate reef. While current levels of ocean warming have allowed the warm‐water fish to extend their ranges into temperate ecosystems at both hemispheres, their foraging performance was reduced at both the cold‐ and warm‐temperate reefs compared to the (sub)tropical reefs. However, at the (warmer) tropicalized reef, the warm‐water fish had higher foraging performance and maintained densities, even under extreme pH reduction, compared to the temperate reef. In contrast, the cool‐water species struggled at the warmer tropicalized and extreme reefs with reduced foraging performance and lower population densities compared to the temperate reef. Contrastingly, the severe heatwave experienced at the temperate reef did not alter the foraging behaviors of either species. We suggest that ocean warming boosts the foraging performance of the range‐extending warm‐water fish and impairs that of their cool‐water competitor at temperate reefs, irrespective of acidification and heatwaves, leading to a shift in dominance hierarchies on temperate reefs. We conclude that warming‐driven increases in foraging performance of the warm‐water species may alleviate foraging limitations and enhance its establishment at its leading range edges under climate change, to the detriment of its cool‐water competitors.

## INTRODUCTION

Anthropogenic warming has facilitated species redistributions to cooler latitudes, higher altitudes, and greater water depths (Parmesan & Yohe, 2003; Pecl et al., 2017), reshaping ecosystem function, structure, and biodiversity in recipient ecosystems (Urban et al., 2012; Vergés et al., 2014). The global redistribution of species often forces range‐shifting and resident species to compete for resources (Alexander et al., 2015), creating novel species interactions (Alexander et al., 2015; Coni, Booth, & Nagelkerken, 2021). How these novel species interactions modify biogeographic boundaries of range‐shifting and resident species under current and future climatic scenarios remains largely unknown, yet is fundamental to forecasting species redistributions under climate change.

To persist in novel environments, animals often modify their behavior (Tuomainen & Candolin, 2011). Behavioral adjustments in novel environments can influence the pace of establishment at higher latitudes, either accelerating or slowing species range extensions (Donelson et al., 2019). Invaders can displace subordinate local species through competition (Milazzo et al., 2013; Sasaki et al., 2024), while local species can also adjust behaviors to coexist with (Coni, Booth, Ferreira, & Nagelkerken, 2021) or limit the establishment of range‐shifting competitors through superior foraging performance (Coni, Booth, & Nagelkerken, 2021) and antagonistic interactions (Twiname et al., 2022). Elevated temperatures can alter behaviors that influence novel competitive interactions between warm‐ and cool‐water competitors in high latitude ecosystems (Mitchell et al., 2023a). Therefore, understanding how novel species interactions are likely to be altered is becoming increasingly important, given the emergence of novel community assemblages in rapidly warming high latitude ecosystems (Soler et al., 2022).

During the summer months, over 150 tropical fish species have been detected extending their ranges toward higher latitudes (Booth et al., 2011; Feary et al., 2014; Vergés et al., 2014). Range extensions of tropical fishes into temperate ecosystems are primarily driven by anthropogenic warming (Pecl et al., 2017), the strengthening of poleward currents (Kumagai et al., 2018; Wu et al., 2012), local environmental conditions (Pinsky et al., 2013), and species‐specific traits (García Molinos et al., 2022). As tropical fishes move into temperate marine ecosystems, they often form novel shoaling interactions with resident temperate fishes (Smith et al., 2018). These novel shoaling interactions can benefit tropical species by reducing mortality, enhancing foraging and growth (Mitchell, Hayes, et al., 2025; Smith et al., 2018), and improving prey access (Paijmans et al., 2020). However, competing temperate fish species may also restrict prey access for tropical fish at higher latitudes (Coni, Booth, & Nagelkerken, 2021), potentially increasing predation upon tropical species (Beck et al., 2016). Meanwhile, the effects of novel tropical–temperate interactions on temperate fish remain poorly understood, with only limited studies providing insight (but see Mitchell et al., 2023a, 2023b; Sasaki et al., 2024). Despite these interactions, tropical fishes currently fail to establish breeding populations in temperate ecosystems (Booth et al., 2011). This is primarily due to winter temperatures often dropping below their minimum thermal temperature tolerances (~18°C; Figueira et al., 2009), leading to mass mortalities. However, with ongoing ocean warming, winter conditions in temperate regions are expected to become more favorable for tropical fish survival and performance (Figueira & Booth, 2010; Mitchell et al., 2023a, 2023b). Thus, research is needed to investigate how future ocean warming will influence interactions between co‐shoaling temperate and tropical fish species in temperate marine ecosystems.

Extreme heatwave events are becoming increasingly intense and frequent in marine ecosystems (Frölicher et al., 2018; Hobday et al., 2016). However, despite our growing knowledge of marine heatwave effects on marine ecosystems (Smale et al., 2019), the impacts of heatwaves on novel species interactions remain unexplored. The thermal stress of marine heatwaves can drive temporary species distribution shifts to deeper waters and higher latitudes (Smale et al., 2019; Smale & Wernberg, 2013). Species range shifts, facilitated by marine heatwaves, could modify existing species interactions or generate new ones between resident and invading species in recipient communities. Indeed, resident species, residing at their warm‐trailing edges, may already be experiencing thermally stressful conditions (Donelson et al., 2019), whereby marine heatwaves push past their thermal upper limits, facilitating range contractions either directly (thermal stress) or indirectly through negative interactions with thermally adapted invaders (Alexander et al., 2015).

Ocean acidification, the increasing dissolution of atmospheric CO_2_ into our oceans (Doney et al., 2009), is both directly and indirectly altering marine ecosystems (Cattano et al., 2020; Nagelkerken & Connell, 2015). It can function as both a stressor and a resource for marine species (Nagelkerken & Connell, 2015), potentially altering species interaction responses detected by standalone ocean warming and marine heatwave effects. Ocean acidification can modify tropical–temperate fish interactions (Mitchell et al., 2022, 2023a), alter resource availability for both range‐extending and resident species (Coni, Nagelkerken, Ferreira, et al., 2021), and indirectly slow tropicalization in marine ecosystems through habitat simplification (Agostini et al., 2021; Cattano et al., 2020). Yet, it remains unclear whether ocean acidification can directly modify novel species interactions in tropicalizing fish communities. Indeed, ocean warming, but not acidification, is likely to produce the greatest effects on the underlying behavioral responses of range‐extending fishes (Mitchell et al., 2023a) involved in novel species interactions (Coni, Booth, & Nagelkerken, 2021). Additionally, how ocean warming, acidification, and increasingly intense marine heatwaves combine to affect species behavioral and physiological responses in rapidly warming ecosystems remains unknown. Therefore, testing the concurrent multi‐stressor impacts (marine heatwaves, ocean warming, and acidification) on competing species is imperative to understanding how novel species interactions will mediate shifts in tropicalizing communities, but has seldom been addressed.

Phenotypic plasticity can allow species to adjust to rapid climate change (Donelson et al., 2019). At their warm‐range edges, resident temperate species that show broad behavioral plasticity may be more resilient to concurrent marine heatwaves and novel tropical competitors than other local species possessing limited phenotypic plasticity (Donelson et al., 2019). In contrast, range‐extending species which display behavioral and physiological plasticity may boost establishment success through adaptive responses to rapidly changing and novel environments at their cold‐leading range edges (Coni, Booth, Ferreira, & Nagelkerken, 2021; Hayes et al., 2024, 2025). Ocean warming could concurrently narrow the phenotypic plasticity of resident temperate species (Rodriguez‐Dominguez et al., 2022) and increase range‐extending species performance in temperate ecosystems in a future ocean (Djurichkovic et al., 2019; Mitchell et al., 2023a). Under multi‐stressor climate change impacts, the capacity for species to modify their behavioral responses could mediate local and invading species persistence in tropicalizing temperate ecosystems. Thus, we must understand how plastic the behavioral responses of range‐extending and resident species are to future climatic conditions and marine heatwave events. This will allow us to determine: (1) the resistance of local species to multiple stressors due to climate change, marine heatwaves, and biological range extensions; and (2) how future climatic conditions and marine heatwaves modify the pace of tropical species range extensions through novel species interactions.

In this study, we assess the foraging performance of co‐shoaling warm‐water and cool‐water fish species responding to multiple climatic stressors (ocean warming, marine heatwaves, and extreme pH reduction) at their respective leading (cold‐edge) and trailing (warm‐edge) range limits in Australia, and at natural analogs of ocean warming and combined warming–extreme acidification conditions in Japan. We measured foraging performance and densities of co‐shoaling warm‐ and cool‐water fishes in situ along three climate change scenarios: (1) across a latitudinal gradient of tropicalization in a global warming hotspot, the East coast of Australia, to address how current levels of ocean warming mediate foraging performance of competing warm‐ and cool‐water fishes; (2) at reef localities in Japan representative of a present‐day “control” temperate reef, a tropicalized temperate reef (where water temperature is higher than the temperate reef), and an extreme temperate reef (where both temperature and CO_2_ levels are higher than both the tropicalized and temperate reefs) to address how future warming and acidification modify warm‐ and cool‐water fish species' foraging performance in temperate reefs; (3) before and during an unprecedented marine heatwave event at a temperate reef in Japan, to evaluate whether marine heatwaves affect the warm‐ and cool‐water fishes' foraging performance observed under present‐day conditions. Both eastern Australia and eastern Japan are among the fastest warming marine regions globally, driven by the strengthening of major western boundary currents, the East Australian Current and the Kuroshio Current, respectively (Vergés et al., 2014). These currents not only warm coastal systems but also facilitate poleward larval transport, leading to parallel tropicalization processes in recipient temperate ecosystems (Vergés et al., 2014). Combining data from these two oceans' warming hotspots allowed us to assess whether behavioral and density responses of warm‐ and cool‐water fishes are consistent across geographic and climate change contexts.

**FIGURE 1:**
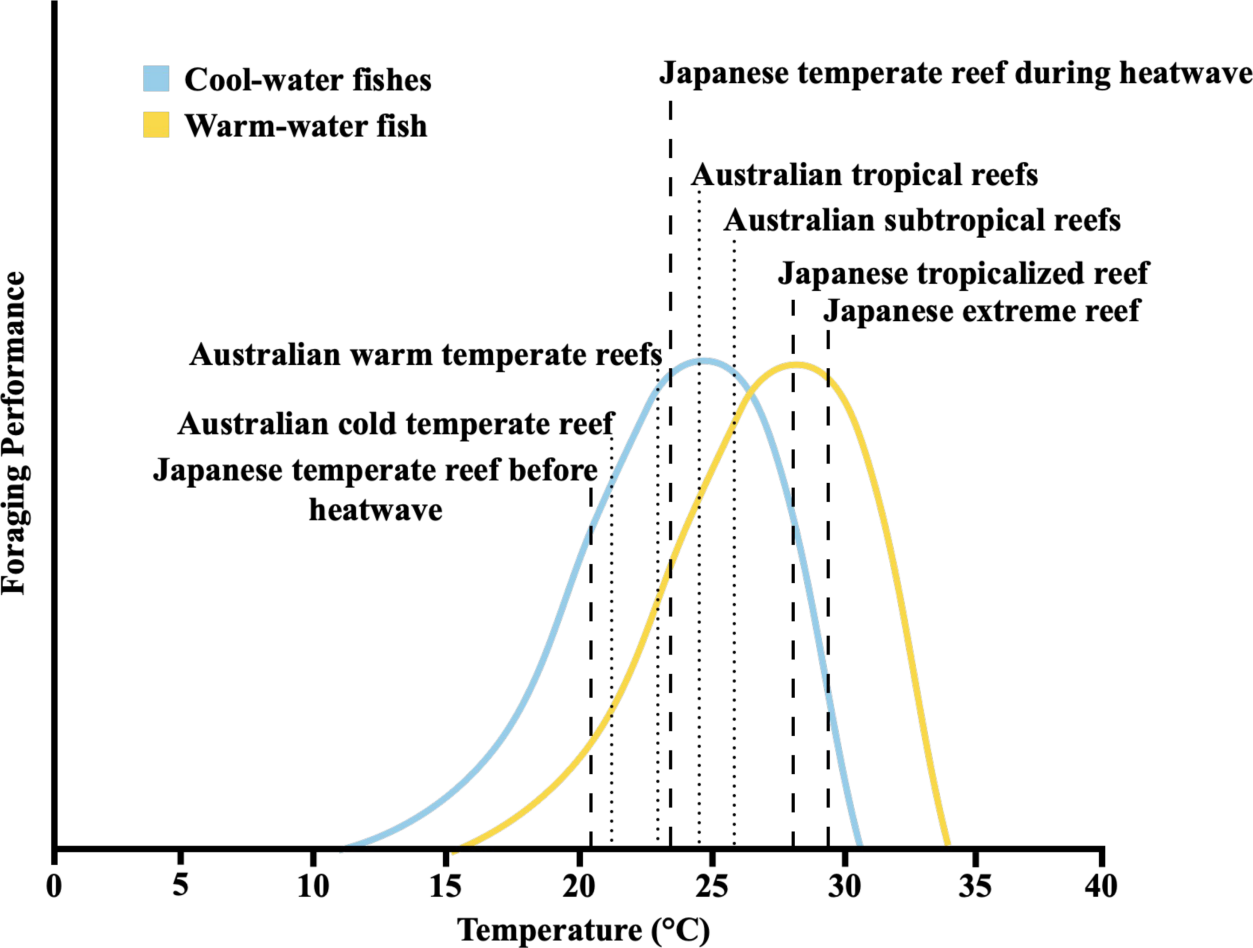
Estimated thermal niche ranges of competing cool‐water (Australia: *Microcanthus joyceae*; Japan: *Microcanthus strigatus*) and warm‐water (*Abudefduf vaigiensis*) fish species compared to sampled seawater temperatures measured during prey release experiments across Australian cold temperate, warm temperate, subtropical, and tropical reefs (dotted lines), and Japanese temperate reef (before and during an early summer marine heatwave), and the tropicalized and extreme reefs during current summer conditions at each reef (dashed lines). Froese (2020) was used to estimate each species' thermal niches from Aquamaps (version 10/2019). The Australian subtropical reefs were sampled in late summer 2021 and were warmer than the tropical reefs, which were sampled in late autumn 2024.

We hypothesize that cool‐water fish are at the peak of their physiological performance in warm‐temperate reefs during summer months (Figure 1) and therefore exhibit superior foraging abilities (higher feeding and aggression rates) than the warm‐water species at these reefs under current ocean warming conditions. Conversely, we expect warm‐water species to show higher foraging performance in a tropicalized reef representative of ocean warming and an extreme reef experiencing combined extreme pH reductions and warming at temperate latitudes than individuals residing in the temperate reef, as higher temperatures boost their physiological functioning (Mitchell et al., 2023b) and pH reductions have limited effects on fish foraging behavior (Cattano et al., 2018). By combining foraging performance data from natural analogs representative of current and future ocean warming and a marine heatwave experiment, our study provides significant insights into the competitive interactions of warm‐water and cool‐water species under heatwaves, and current and future ocean warming and acidification, enhancing our understanding of species redistributions in a future ocean.

## MATERIALS AND METHODS

### Ethics statement

All experiments were performed under animal ethics approvals S‐2017‐002 and S‐2023‐043 (University of Adelaide), ETH17‐1117 (University of Technology Sydney), and GBRMPA permit: G20/43958.1, and followed both universities' animal ethics guidelines. Prey release experiments and fish density surveys were conducted under Shizuoka Prefecture permit number 5‐10 (2023) and Tokyo Prefecture permit number 5‐11 (2023).

### Study species

We selected one model species with a tropical affinity (hereafter referred to as the warm‐water species): the most common tropical range‐extending species along the Australian east coast, *Abudefduf vaigiensis* (Booth et al., 2011), and two subtropical species (hereafter referred to as the cool‐water species): *Microcanthus strigatus* in Japan and *Microcanthus joyceae* in Australia, both of which form shoals with *A. vaigiensis*. The warm‐water species is commonly found in warm Indo‐Pacific reefs (Booth et al., 2007, 2011) but also appears as a vagrant each summer along the southeastern Australian temperate coastline (34–37° S; Booth et al., 2007) and at similar latitudes in Japan during the northern hemisphere summer months (Cattano et al., 2020). In Australia, the cool‐water species (*M. joyceae*) occurs in subtropical and temperate Australian reefs from southern Queensland to southern New South Wales (Tea & Gill, 2020), while *M. strigatus* occurs from southern China to central Japan (~38° N; Tea & Gill, 2020). Both focal cool‐water species were considered a single species up until a recent systematic reappraisal (Tea & Gill, 2020). The warm‐water species has coexisted with both cool‐water fish species for extended periods of time (>10 years) at Australian subtropical reefs (Booth et al., 2007, 2011) and at Japanese tropicalized and extreme reefs (Cattano et al., 2020), than at temperate reefs, in Australia and Japan, where they only co‐occur during warmer summer and autumn months (Booth et al., 2007, 2011).

### Study locations

#### Australian latitudinal gradient

**FIGURE 2:**
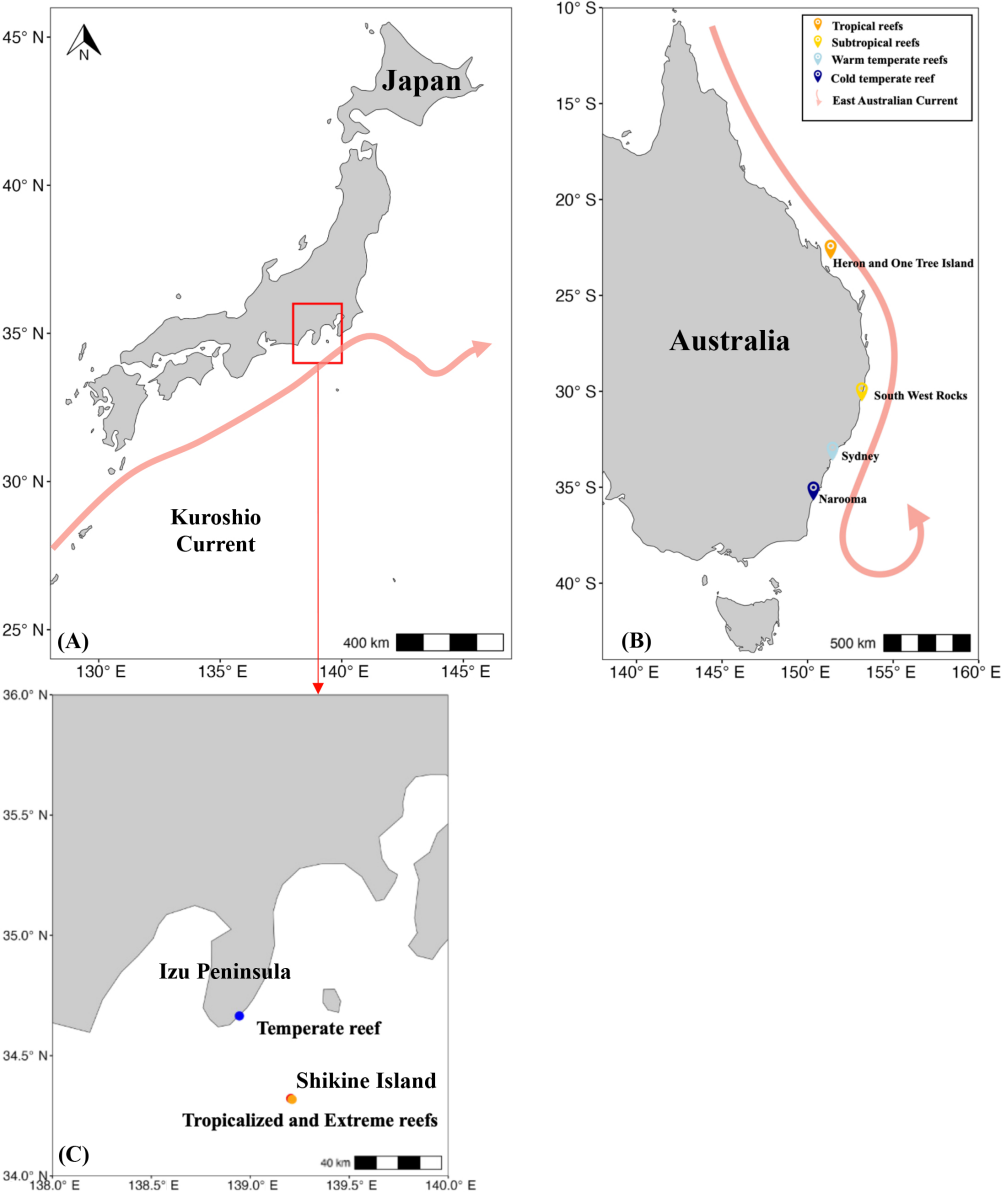
Maps of (A) Japanese and (B) Australian reefs connected by the Kuroshio and East Australian Currents, respectively. (C) The Japanese temperate reef (blue dot) located on the coast of the Shimoda, tropicalized reef (orange dot) and extreme reef (red dot) sampled located on the coast of Shikine Island. Tropicalized and extreme reefs are annually warmer (+1°C), on average, than the warm temperate reef in Japan due to (B) the warm‐water Kuroshio Current. Arrows indicate direction of the Kuroshio and East Australian currents.

We conducted visual fish abundance surveys and in situ prey release experiments at eight sites spanning tropical to cold temperate reefs along the eastern Australian coast, a global warming hotspot (Figure 2). Surveys were conducted during the austral summers of 2017, 2018, and 2024, when tropical fish abundances peak in temperate reefs (January–May). The tropical reefs (Heron Island and One Tree Island in the Great Barrier Reef) represent the core range of warm‐water fish and the warm‐trailing edge of cool‐water species. These sites were characterized by wave‐sheltered barren rocky habitats. The subtropical reefs (two sites at South West Rocks) mark the southernmost breeding region for range‐extending warm‐water fish, where mean winter seawater temperatures (Table 1) remain above their lower thermal tolerance (~18°C; Figueira et al., 2009). The benthic environment at these sites consisted of either sparse rocky oyster reefs (site 1) or bare rocky substratum (site 2). The warm temperate reefs were located around Sydney and included three tropicalization hotspots (Booth et al., 2007, 2011): Little Manly, Shelly Beach, and Narrabeen. The benthos at Little Manly and Shelly Beach consisted of macroalgae (e.g., kelp and sargassum), crustose coralline algae, turf algae, rocky substrates, and urchin barrens, whereas Narrabeen was characterized by bare rock, oyster reef, and turf algae. The cold temperate reef at Narooma was the coldest reef studied (Table 1) and the most novel habitat for range‐extending warm‐water fishes.

**TABLE 1:** Seawater chemistry of the sampled reefs in Australia and Japan.

Metric	Temperature (°C)	pH	Salinity (ppt)	pCO_2_ (kPa)
Japan reefs				
Temperate reef before global heatwave (17 July 2023)	20.78 (±0.03)	8.088 (±0.005)	33.98 (±0.03)	0.0505
Temperate reef during global heatwave (30 July 2023)	23.80 (±0.04)	8.070 (±0.009)	34.05 (±0.06)	0.0539
Tropicalized reef during global heatwave (27 July 2023)	27.48 (±0.49)	8.189 (±0.06)	34.40 (±0.06)	0.0393
Extreme reef during global heatwave (25 and 28 July 2023)	28.91 (±0.06)	7.661 (±0.09)	34.37 (±0.03)	0.1635
Australian reefs				
Tropical reefs	25.2 (±0.00)	…	…	…
Subtropical reefs	25.7 (±0.00)	…	…	…
Warm temperate reefs	23.0 (±0.00)	…	…	…
Cold temperate reef	21.4 (±0.00)	…	…	…

*Note*: Values are reported as mean (±SE). Partial pressure of CO₂ (*p*CO_2_) is reported in kilopascals (kPa). *p*CO_2_ values were calculated using mean total alkalinity for each reef, sourced from Agostini et al. (2021). For the temperate reef, total alkalinity was estimated from seawater pumped from ~5 m depth at Shimoda Marine Research Center, University of Tsukuba (Agostini et al., 2021). Seawater chemistry for Japanese reefs was measured immediately after behavioral video recordings. Water temperature data for subtropical, warm‐temperate, and cold‐temperate reefs in Australia were obtained from Coni, Booth, and Nagelkerken (2021) and reflect late summer to early autumn conditions. An ellipsis indicates that pH, salinity, and *p*CO_2_ were not recorded at Australian reefs during the 2017, 2018, and 2024 sampling campaigns.

#### Tropicalized and extreme reef analogs in Japan

In Japan, we carried out visual fish abundance surveys and in situ prey release experiments at three shallow nearshore reefs at 0.5–3 m depth (Figure 2) during July 2023. One reef is characteristic of a present‐day temperate marine ecosystem (hereafter referred to as “temperate reef”), where the warm‐ and cool‐water species co‐occur only during summer and autumn months, and is located off the Izu Peninsula (34.665051, 138.945387). The temperate reef is characterized by a mosaic of macroalgae covering the rocky substrate (Appendix S1: Figure S1). The two other reefs are naturally warmer than the “temperate reef” and are located ~50 km from the “temperate reef” along the shoreline of Shikine‐jima, Japan. One reef is an analog for a tropicalized temperate reef (hereafter: “tropicalized reef” [34.318142, 139.211097]), while the second reef experiences a higher level of warming as well as acidification from a natural carbon dioxide seep, reaching *p*CO_2_ levels beyond SSP5‐8.5 2100 climate projections (Table 1), making it an analog for extreme pH reduction and warming (hereafter: “extreme reef” [34.321872, 139.203848]). The tropicalized reef is characterized by turf, corals, and some macroalgae, and the extreme reef is characterized by homogeneous turf‐covered rocky substrate (Agostini et al., 2021; Harvey, Kon, et al., 2021; Appendix S1: Figure S1). At these two reefs, warm‐ and cool‐water species coexist throughout the year (Cattano et al., 2020), providing a unique opportunity to examine how ocean warming alone, and combined with extreme acidification, could modify novel foraging interactions in a tropicalizing fish community. At all three reefs, juvenile warm‐water and cool‐water fish species were observed to co‐occur in shoals at 0.5–3 m depths, near rocky structures.

### Marine heatwave event in Japan

Following Hobday et al. (2016), we defined a marine heatwave event as “a prolonged discrete anomalously warm event that last for five or more days, with temperatures warmer than the 90th percentile based on a 30‐year historical baseline period.” During the study period, category II marine heatwaves were experienced at the temperate, tropicalized, and extreme reefs in Japan (Appendix S1: Figure S2). Marine heatwave data for reefs in Japan were extracted from Marineheatwavetracker.org. At the temperate reef, a strong marine heatwave event with a cumulative intensity of 189.24 degree‐days and maximum intensity of 3.39 (in degrees Celsius) above the 30‐year mean temperature lasted for 78 days.

We conducted prey release experiments before and during the marine heatwave event only at the temperate reef. At the tropicalized and extreme reefs, a marine heatwave with a cumulative intensity of 78.87 degree‐days and a maximum intensity of 3.51 (in degrees Celsius) above the 30‐year mean temperature lasted for 30 days. We performed prey release experiments during but not before the marine heatwave event at the tropicalized and extreme reef localities.

### Seawater carbonate chemistry

Seawater carbonate chemistry at the temperate, tropicalized, and extreme reefs has been monitored over multiple years (Agostini et al., 2015, 2018, 2021; Cattano et al., 2020; Harvey, Allen, et al., 2021). Salinity, temperature, and pH were measured (*n* = 3–4 per sampling period per reef) on days when prey release experiments were conducted. Sensors for salinity, temperature, and pH were calibrated 3 days prior to measurements at each reef locality following the manufacturer's instructions. *p*CO₂ values for the Japanese reef localities were computed using CO2SYS (Pierrot et al., 2006) for Excel, based on total alkalinity data from Agostini et al. (2015, 2021). The *p*CO₂ calculations used constants from Mehrbach et al. (1973), refit by Dickson and Millero (1987). At the Australian reefs (excluding the tropical reefs), temperatures were recorded concurrently with prey release experiments using Hobo Pendant 64K Temp‐Alarm continuous data loggers. For the tropical reefs, maximum daily temperature data for sampling days at One Tree Island were obtained from the Australian Institute of Marine Science Data Repository (AIMS, 2024).

### In situ prey release experiment

In Australia and Japan, we (1) quantified aggressive interactions between the focal cool‐water and warm‐water species, and (2) tested their foraging performance based on seven foraging and interaction proxies as a function of local seawater temperature and pH conditions.

An in situ prey release experiment was conducted to attract the two focal fish species and stimulate species interactions (Appendix S1: Figure S1), following the methods of Coni, Booth, and Nagelkerken (2021). At the Australian reefs, prey release experiments were performed at subtropical (*n* = 40), warm temperate (*n* = 44), and cold temperate reefs (*n* = 38) during the Austral summers of 2017 and 2018. In May 2024, prey release experiments (*n* = 9) were performed at Australian tropical reefs (Heron Island and One Tree Island). At the Japanese reefs, prey release experiments were conducted at the temperate reef on 17 (before heatwave: *n* = 8) and 30 (during heatwave: *n* = 8) July 2023, respectively, and at the tropicalized (*n* = 8) and extreme reef (*n* = 9) on 25, 27, and 28 July 2023, respectively. The prey release experiment involved dispersing a mixture of 60 mL of seawater and ~1.25 g of dead brine shrimp (*Artemia*). This mixture was administered using a 60‐mL plastic syringe connected to a transparent tube (2.5 mm in diameter, 1 m in length) fixed to a 1‐kg lead weight. The prey release setup was placed on the reef benthos ~50 cm from a GoPro 7 Silver camera, which was secured to the substrate and positioned toward the prey release point (Appendix S1: Figure S1). The prey release setup was specifically designed to enhance observations of juvenile tropical fish, typically less than 5 cm in total length, and has been successfully employed in previous studies for fish of similar size (Coni, Booth, & Nagelkerken, 2021).

At the beginning of each experiment, 30 mL of the syringe's contents were released. Once the prey had been consumed or dispersed (~5 min), the remaining 30 mL was released. Two observers conducted the experiment while snorkeling, moving more than 5 m away after the initial release and briefly returning for the second release. A 2‐min acclimation period was included before prey release. Experiments were recorded at 1080p resolution and 25 frames per second.

Juveniles of the two cool‐water and one warm‐water species are site‐attached (Coni, Booth, & Nagelkerken, 2021), reducing the likelihood of recording the same individual across replicates. To ensure independent observations, prey release experiments were randomly conducted at least 3 m apart, targeting different sheltering shoals of focal warm‐ and cool‐water species.

### Fish behavior video analyses

All seven focal fish behaviors were analyzed using QuickTime Player on a desktop computer. Video recordings captured up to 5 min of foraging behavior, but as fish moved in and out of view, focal observation times ranged from 20 s to 5 min (average ~ 2.5 min). Short observation periods have been shown to provide representative estimates of these behaviors (Beck et al., 2016; Coni, Booth, & Nagelkerken, 2021). To avoid pseudoreplication, we recorded the behaviors of one cool‐water species (Japan: *M. strigatus*; Australia: *M. joyceae*) and one warm‐water species (*A. vaigiensis*) per video (Appendix S1: Table S1 provides sample sizes for each species at reefs in Japan and Australia).

We quantified seven behaviors for each focal fish in the video recordings (Appendix S1: Table S2), following the methodological framework described in Coni, Booth, and Nagelkerken (2021). (1) Prey attraction time was recorded as the duration (in seconds) from prey release until the fish swam to the prey source (tube end) and took its first bite. (2) Minimum distance to prey was determined as the closest horizontal approach (in centimeters) to the prey release point, estimated visually using a ruler on the computer screen; if the fish did not approach along a horizontal plane, this measure was not recorded. (3) Prey inspection rate was quantified by counting the total number of times a focal fish moved within five body lengths of the prey release site immediately after prey release. (4) Bite rate was quantified as the total number of bites successfully taken at prey items released from the prey release point. (5) Retreat rate represented the total number of instances where a fish approached within five body lengths of the prey release site but then abruptly retreated. (6) Chasing rate was counted as the total number of times the focal fish aggressively swam toward a heterospecific fish, attempting to displace it from the prey. (7) Escaping rate measured the total number of times the focal fish fled from an aggressive fish. Behaviors recorded as continuous counts—including prey inspection, bite, retreat, chasing, and escaping—were standardized as rates per unit time (e.g., in number of bites per second).

### Fish density surveys

Fish densities at each reef were quantified using 10‐m belt transects. A snorkeler swam along each 10‐m transect, counting all individuals of target fish species within a 2‐m width on both sides of the transect tape, covering a total survey area of 40 m^2^ per transect.

In Australia, the densities of focal warm‐ and cool‐water fish species were estimated at the tropical (*n* = 20 transects), subtropical (*n* = 15), warm temperate (*n* = 40), and cold temperate (*n* = 31) reefs in Austral summer and autumn of 2021. In Japan, the densities of the two focal fish species were estimated at the temperate (*n* = 5), tropicalized (*n* = 6), and extreme (*n* = 5) reefs between 27 and 30 July 2023.

### Statistical analyses

We tested for differences in the overall foraging performance of each species across reef climate scenarios using permutational multivariate ANOVA (PERMANOVA). This approach allowed for the inclusion of multiple behavioral metrics per species and tested the effect of climate scenarios on the overall behavioral repertoire of the cool‐ and warm‐water species. We used PERMANOVA to assess the effect of each climate scenario across the Japanese natural analogs (two‐way PERMANOVA: fixed factor “Species”: “warm‐water” vs. “cool‐water,” and three levels of fixed factor “Reef”: “temperate reef,” “tropicalized reef,” “extreme reef”), and the Australian tropicalization analogs (three‐way PERMANOVA: fixed factor “Species”: “warm‐water” vs. “cool‐water,” and four levels of fixed factor “Reef”: “tropical,” “subtropical,” “warm temperate,” and “cold temperate” reef) on standardized log (*X* + 1)‐transformed data using Bray–Curtis resemblance matrices. This approach allowed us to evaluate cool‐ and warm‐water species shifts in foraging performance across climate scenarios, consistent with our hypothesis that each species' behavioral performance would diverge across thermal gradients. We selected PERMANOVA for its ability to handle multivariate, nonindependent ecological response variables without requiring normality or equal variances, and therefore suitable for our unbalanced sampling design and multivariate behavioral data (Anderson et al., 2008).

To investigate marine heatwave impacts on cool‐ and warm‐water fish interactions and behaviors, we ran a secondary analysis on the Japanese temperate reef only, where foraging data were collected before and during an unprecedented marine heatwave event. We used a two‐way PERMANOVA (fixed factors: Species: “warm‐water” vs. “cool‐water” and “Heatwave”: “before” vs. “during”). To account for multiple comparisons on the Japan natural analog PERMANOVA data, a Bonferroni correction was applied to the secondary analysis on the temperate reef PERMANOVA (*p* < 0.025).

PERMANOVA tests revealed significant effects of one or more fixed factors on foraging performance in Japan and Australia (Appendix S1: Tables S3 and S4). To identify which behaviors contributed most to the observed multivariate differences across reefs and species, we conducted Similarity Percentage (SIMPER) analyses based on Bray–Curtis dissimilarities. We then visualized multivariate patterns in foraging performance using nonmetric multidimensional scaling (nMDS) plots, generated from Bray–Curtis resemblance matrices of log‐transformed and standardized data. nMDS ordinations were constructed separately for each species group to explore behavioral variation among reefs in Japan and Australia, with vectors overlaid to indicate the relative influence of each behavior on ordination space.

We then conducted post hoc univariate permutational ANOVAs to identify the specific behaviors driving reef or species differences. All univariate behavioral analyses were performed on log (*X* + 1)‐transformed data using Euclidean resemblance matrices. For significant effects (*p* < 0.05) detected in the main ANOVA tests, pairwise tests compared the respective means. Differences in focal warm‐water and cool‐water fish densities at the Australian and Japanese natural analogs were tested using two‐way permutational ANOVAs with “Reef” and “Species” as fixed factors. Behavior and density data were analyzed using PRIMER v7 and PERMANOVA+ (Anderson, 2001). Permutational ANOVA only assumes that samples are exchangeable under the null hypothesis, which holds for our fully randomized design (Anderson et al., 2008). We selected this method due to its robustness to heterogeneity in variance, which is common in ecological data (Anderson et al., 2008).

**FIGURE 3:**
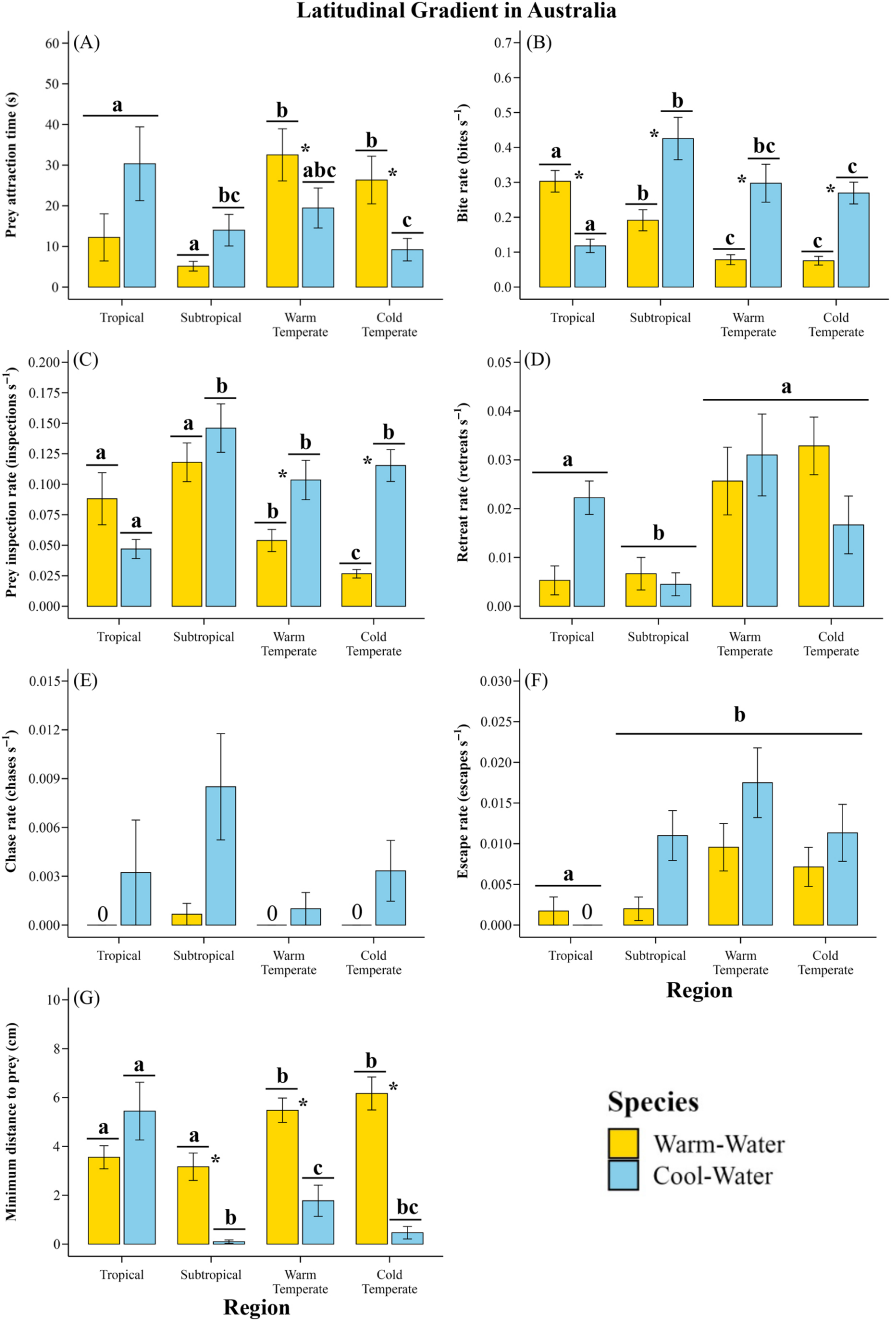
Foraging performance proxies (mean ± SE) for warm‐water (*Abudefduf vaigiensis*) and cool‐water (*Microcanthus joyceae*) fishes across a ~1300‐km latitudinal gradient from tropical to cold temperate reefs. Food acquisition metrics include (A) prey attraction time, (B) bite rate, (C) prey inspection rate, (D) retreat rate, and (G) minimum distance to prey. Aggressive interactions are represented by (E) chasing rate and (F) escape rate. Significant differences between reefs within each species are indicated by unique letters above bars (*p* < 0.05; see Appendix S1: Tables S9–S15). “NS” denotes no significant differences (*p* > 0.05). Significant differences between warm‐water and cool‐water species within reefs are indicated by an asterisk (see Appendix S1: Tables S9–S15).

**FIGURE 4:**
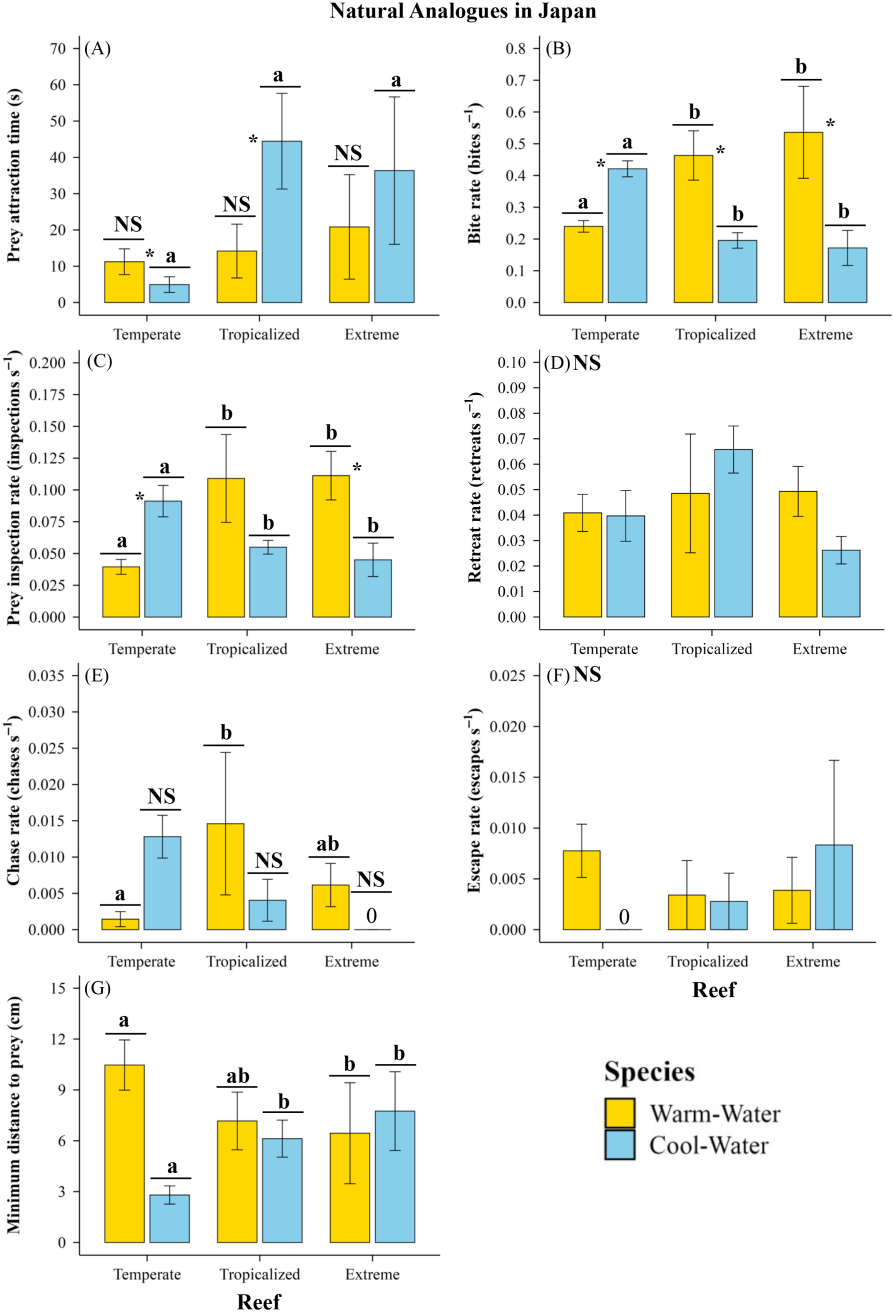
Foraging performance proxies (mean ± SE) for warm‐water (*Abudefduf vaigiensis*) and cool‐water (*Microcanthus strigatus*) fishes across a natural analog for ocean warming, extreme pH reduction, and warming. Food acquisition metrics include (A) prey attraction time, (B) bite rate, (C) prey inspection rate, (D) retreat rate, and (G) minimum distance to prey. Aggressive interactions are represented by (E) chasing rate and (F) escape rate. Significant differences between reefs within each species are indicated by unique letters above bars (*p* < 0.05; see Appendix S1: Tables S17–S20 and S22–S24). “NS” denotes no significant differences (*p* > 0.05). Significant differences between warm‐water and cool‐water species within reefs are indicated by an asterisk (see Appendix S1: Tables S17–S20 and S22–S24).

**FIGURE 5:**
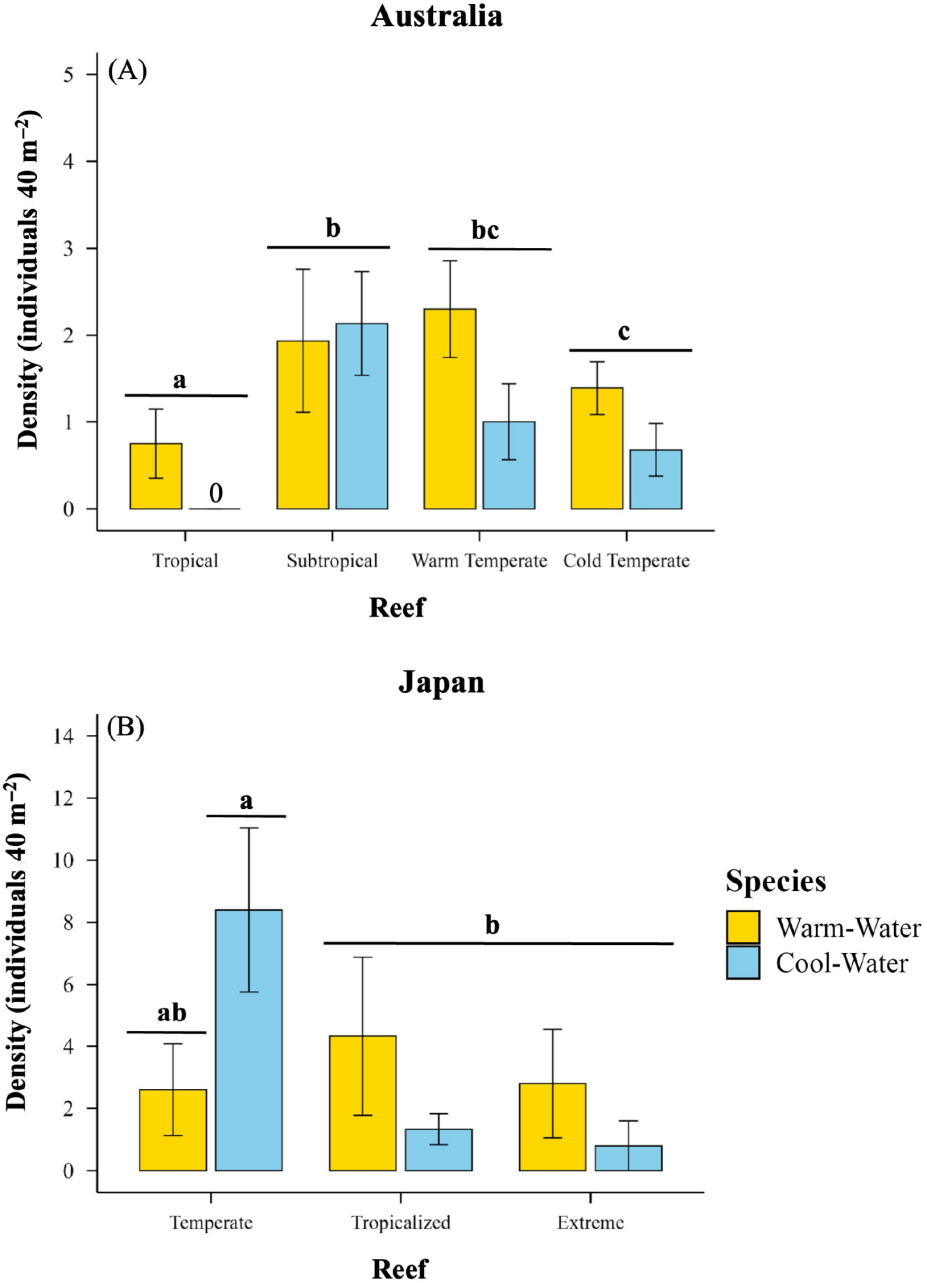
Densities (mean ± SE) of warm‐water (*Abudefduf vaigiensis*) and cool‐water (Australia: *Microcanthus joyceae*; Japan: *Microcanthus strigatus*) focal fish species across the (A) Australian latitudinal gradient from tropical to cold temperate reefs and (B) Japanese reefs representative of present‐day, temperate, tropicalization, and extreme reef localities (see Figure 2). Letters above bars indicate significant differences between reefs within species (*p* < 0.05; see Tables S16 and S21). See Appendix S1: Tables S16 and S20 for statistical outputs reported in Figure 5.

Figures 2, 3, 4, 5 were created using the package “*ggplot2*” (Wickham, 2016) in *R* version 4.4.0 (R Core Team, 2024).

Foraging behavior data for subtropical, warm‐temperate, and cold‐temperate reefs has been previously reported in Coni, Booth, and Nagelkerken (2021). Here, we reuse data for Australian subtropical, warm‐temperate, and cold‐temperate reefs in a new analysis which includes data for warm‐water and cool‐water species at tropical reef localities in Australia in our statistical analysis.

## RESULTS

### Key behaviors driving foraging dissimilarity across reefs

Across both Australian and Japanese reefs, bite rate, prey attraction time, minimum distance to prey, and prey inspection rate were the key behaviors driving multivariate dissimilarities in foraging performance among reef types for cool‐ and warm‐water fish species (Appendix S1: Figure S3, Tables S5–S8). In Australia, these four behaviors together explained over 90% of the behavioral dissimilarity between tropical and temperate reef types for cool‐ and warm‐water fish species (Appendix S1: Figure S3). In Japan, they explained >86% of dissimilarity between the temperate and the tropicalized and extreme reefs (SIMPER; Appendix S1: Tables S5–S8).

### Warm‐water fish showed improved foraging performance at warmer reefs compared to cooler reefs

In Australia, the warm‐water species' overall foraging performance shifted significantly between the (sub)tropical and cold‐temperate reefs (Table 2; Appendix S1: Figure S3A, Table S3).

**TABLE 2:** Significant behavioral and fish density patterns (*p* < 0.05; Appendix S1: Tables S3–S20) for the warm‐water fish (*Abudefduf vaigiensis*) at their novel leading distributions (cold‐ and warm‐temperate reefs) compared to their core ranges (tropical reefs) and the cool‐water fish (*Microcanthus joyceae*) at their trailing range edge (tropical reefs) compared to their core ranges (warm‐temperate reefs) in Australia. In Japan, significant behavioral and fish density patterns (*p* < 0.05) for focal warm‐water (*A. vaigiensis*) and cool‐water (*Microcanthus strigatus*) at the tropicalized reef in comparison to the temperate reef are shown under the “Natural Analogs in Japan” subheading.

Affinity	Prey attraction time	Bite rate	Prey inspection rate	Retreat rate	Chase rate	Escape rate	Minimum distance to prey	Density
Latitudinal gradient in Australia								
Warm‐water	+	−	−	=	=	+	+	+
Cool‐water	+	−	−	=	=	−	+	−
Natural analogs in Japan								
Warm‐water	=	+	+	=	+	+	=	=
Cool‐water	+	−	−	=	=	=	+	−

*Note*: Symbols indicate significant increases (+), decreases (−), or no change (=) in behaviors (prey attraction time, bite rate, prey inspection rate, retreat rate, chase rate, escape rate, and minimum distance to prey) and densities for each species across these distributional shifts. Additionally, significant behavioral and fish density patterns (*p* < 0.05) for focal warm‐water (*A. vaigiensis*) and cool‐water (*Microcanthus strigatus*) at the tropicalized reef in comparison to the temperate reef in Japan are shown under the “Natural Analogs in Japan” subheading. Symbols represent significant increases (+), decreases (−), or no change (=) in behaviors and fish densities at the tropicalized and extreme reefs relative to the temperate reef for both species. The marine heatwave analysis conducted at the Japanese temperate reef is not included here, as the initial multivariate ANOVA (MANOVA) revealed no significant effects (*p* > 0.05) on the overall behavioral repertoire of either focal species. As a result, permutational ANOVAs for each behavior were not conducted.

At the cold temperate reef, the warm‐water fish had 38%–60% lower prey inspection rates and 45%–55% lower bite rates than at subtropical and tropical reefs (Figure 3B,C; Table 2; Appendix S1: Tables S9 and S10), while their prey attraction time, retreat rates, escape rates, and minimum distance to prey (Figure 3A,D,F,G; Table 2; Appendix S1: Tables S11–S14) were 43%–89% higher in the cold‐ and warm temperate reefs than the subtropical and tropical reefs (except tropical vs. warm temperate reefs for prey inspection rates, tropical vs. cold‐ and warm temperate reefs for retreat rates, and subtropical vs. cold temperate reefs for escape rates; Appendix S1: Tables S11–S13). The warm‐water fish's chase rates did not change across reefs (Figure 3E; Appendix S1: Table S15); however, their densities at the cold temperate reef were 39% lower than at the subtropical reefs but 85% higher than at the tropical reefs (Figure 5A; Appendix S1: Table S16).

In Japan, the warm‐water species' overall foraging performance differed significantly between the temperate and extreme reefs, but not between the temperate and tropicalized reefs (Appendix S1: Figure S3C, Table S4).

In Japan, the warm‐water fish was 38%–46% further from prey, had 64%–65% lower prey inspection rates, 48%–55% lower bite rates, and 77%–90% lower chase rates at the temperate reef than at the tropicalized and extreme reefs (Figure 4B,C,E,G; Appendix S1: Tables S17–S20), except for comparisons between tropicalized and temperate reefs for minimum distance to prey, and between extreme and temperate reefs for chase rates (Appendix S1: Tables S18 and S20). The densities, prey attraction times, retreat rates, and escape rates of the warm‐water fish did not significantly differ between reefs in Japan (Figures 4A,D,E and 5B; Table 2; Appendix S1: Tables S21–S24).

The warm‐water species behavior remained unaffected by the marine heatwave at the temperate reef (Appendix S1: Table S25).

### The cool‐water fishes showed superior foraging at cooler reefs than warm reefs

In Australia, the cool‐water species' overall foraging performance shifted significantly from the tropical reefs compared to the subtropical and temperate reefs (Table 2; Appendix S1: Table S3), as well as between warm temperate and subtropical reefs (Appendix S1: Figure S3B, Table S3).

At the Australian cold‐ and warm‐temperate reefs, the cool‐water fish species had 66%–85% faster prey attraction times, was 41%–52% closer to prey, and had 83%–88% higher prey inspection rates, 136%–143% higher bite rates, and 102%–140% higher escape rates, respectively, compared to the tropical reef (Figure 3A–C,F,G; Appendix S1: Tables S9–S11, S13, and S14). The cool‐water species also showed 76%–127% higher retreat rates and 29%–49% lower densities at the tropical and cold‐temperate reefs compared to the subtropical reef (Figures 3C and 5A; Appendix S1: Tables S12 and S16).

In Japan, the cool‐water species' overall foraging performance differed significantly between the temperate and the tropicalized and extreme reefs (Appendix S1: Figure S3D, Table S4).

In Japan, the cool‐water fish species showed 86%–89% faster prey attraction times, was 54%–64% closer to prey, and had 115%–145% higher bite rates, 66%–103% higher prey inspection rates, and >216% higher chase rates at the temperate reef than at the warming and extreme reefs, respectively (Figure 4A–C,E,G; Appendix S1: Tables S17–S20 and S22). At the temperate reef, cool‐water species densities were also 533% and 960% higher than at the tropicalized and extreme reefs, respectively (Figure 5B; Appendix S1: Tables S21).

The cool‐water species' behavior was unaffected by the marine heatwave at the temperate reef (Appendix S1: Table S25).

### Foraging dominance shifted from cool‐ to warm‐water fish at warmer reefs

In Australia, for six of the seven behaviors, the cool‐water fish had higher foraging performance than the warm‐water fish at either of the subtropical, warm‐temperate, or cold‐temperate reefs: higher bite rate (Figure 3B; Appendix S1: Table S10), prey inspection (Figure 3C; Appendix S1: Table S9), and escape rates (Figure 3F; Appendix S1: Table S13), as well as faster prey attraction times (Figure 3A; Appendix S1: Table S11). However, the warm‐water species had higher densities than the cool‐water across all reefs in Australia (Figure 5A; Appendix S1: Table S16), and higher bite rates than the cool‐water fish in the tropical reefs (Figure 3B; Appendix S1 Table S10).

In Japan, the warm‐water fish had faster prey attraction times (Figure 4A; Appendix S1: Table S22) and higher bite rates (Figure 4B; Appendix S1: Table S19) than the cool‐water fish at the tropicalized reef and showed higher prey inspection rates at the extreme reef (Figure 4C; Appendix S1: Table S18).

At the temperate reef in Japan, the behaviors between the warm‐water and cool‐water species did not differ before or during the marine heatwave event (Appendix S1: Table S25).

## DISCUSSION

Here, we show that ocean warming affects a suite of behaviors that mediate the foraging performance of competing warm‐ and cool‐water fishes in rapidly tropicalizing marine ecosystems in the northern and southern hemispheres. In Australia, the studied warm‐water fish had compromised foraging performance at its cold‐temperate range edge, compared to subtropical and tropical reefs within its core range. Reductions in bite and inspection rates, and increased distance to prey, prey attraction time, escape rates, and retreat rates may have been caused by cold stress (Figueira & Booth, 2010; Mitchell et al., 2023a) and the environmental novelty encountered in temperate reefs (Coni et al., 2022). Additionally, the co‐shoaling cool‐water species demonstrated higher foraging performance—including higher chase, bite, and inspection rates, and shorter prey attraction time and distance to prey—than its warm‐water counterpart in warm‐ and cold‐temperate reefs in Australia, potentially limiting the ability of warm‐water fish to establish at temperate latitudes (Coni, Booth, Ferreira, & Nagelkerken, 2021; Coni, Booth, & Nagelkerken, 2021; Coni, Nagelkerken, Ferreira, et al., 2021).

However, at the tropicalized and extreme reefs, near future levels of ocean warming boosted the warm‐water fish species' foraging performance through increased bite rates, prey inspection rates, reduced distance to prey, and increased chase rates of cool‐water competitors compared to the temperate reef, at their leading distributions in Japan. Conversely, the cool‐water fish's foraging performance decreased (with reduced bite and prey inspection rates, and increased prey attraction time and distance to prey) at the tropicalized and extreme reefs, likely due to suboptimal warm water temperatures. Thus, we suggest that ocean warming can drive a reversal of foraging performance between these two species—and possibly many other species as well—as temperate reef ecosystems become more thermally favorable for warm‐water fish and less suitable for their cool‐water competitors (Figure 1). While foraging ability is a key ecological trait linked to competitive dominance (Tuomainen & Candolin, 2011), improved foraging performance does not directly imply increased individual fitness, such as higher survival, growth, or reproductive success in tropical fish residing in temperate ecosystems. Nonetheless, we conclude that ocean warming weakens the foraging superiority of cool‐water fishes on tropicalized reefs, thereby favoring warm‐water species. Consequently, our findings support the theory that rising temperatures will accelerate the tropicalization of temperate reefs in the near future.

While ocean warming may directly boost the foraging performance of warm‐water fishes at their leading ranges, our results also show that warming can indirectly alleviate competition by reducing the densities of resident competitors on temperate reefs. In Japan, lower cool‐water fish densities were observed at the warmer tropicalized and extreme reef localities compared to the temperate reef, where temperatures approached that of the trailing edge of the cool‐water species' thermal range (Froese, 2020). Currently, cool‐water species often maintain competitive superiority over warm‐water counterparts in novel leading range distributions due to larger body sizes (Sasaki et al., 2024; Smith et al., 2018), higher densities (Coni, Booth, & Nagelkerken, 2021), and greater thermal suitability (Coni et al., 2022). We suggest that as ocean warming intensifies, it may concurrently reduce both the foraging performance and densities of cool‐water species on temperate reefs, thereby relieving warm‐water species of competition and accelerating the tropicalization of temperate reef fish communities in the near future.

Marine heatwaves can impose acute thermal stress on reef fishes (Bernal et al., 2020; Van Wert et al., 2024), yet their effects depend on whether resulting temperatures surpass a species' thermal tolerance limits (Alfonso et al., 2021). We demonstrate that the unprecedented northern‐hemisphere marine heatwave of 2023 did not affect the foraging performance of either the warm‐ or cool‐water fish species at the temperate reef in Japan. At this reef, temperatures during the marine heatwave fell within the optimal thermal range for both the warm‐water and cool‐water species studied (Froese, 2020). Given that the marine heatwave temperatures occurred at the leading range for the warm‐water species and at the core range of the cool‐water species, it is unlikely that either species was behaviorally affected by an acute +3°C increase in temperature. However, future warming may create thermally stressful conditions for the cool‐water species, whereby concurrent marine heatwaves may push temperatures past their upper thermal limits (Donelson et al., 2019), especially at their trailing edges. We conclude that marine heatwaves currently have limited behavioral impacts on competing warm‐water and cool‐water fishes at temperate reefs where seawater temperatures remained below both species' critical thermal thresholds.

Importantly, our study shows that extreme pH reductions beyond SSP5 projections did not mediate shifts in the foraging performance of competing cool‐ and warm‐water fishes residing in temperate reefs. At the extreme reef in Japan, the behaviors of both cool‐ and warm‐water fishes remained largely unaffected compared to the tropicalized and temperate reefs. We suggest that both studied fish species may be physiologically tolerant to ocean acidification (which represents much less extreme pH reductions than at our extreme reef) through modified acid–base regulation (Cattano et al., 2018) and plastic molecular responses (Petit‐Marty et al., 2021). Tolerance to extreme pH reductions may partly aid these species under future climates, yet ocean warming and more frequent marine heatwaves still pose significant threats (Hobday et al., 2016), particularly at the species' warm range edges (Donelson et al., 2019). Thus, we conclude that while both species may be tolerant to extreme pH reductions in their studied ranges, ocean warming will play a more dominant role in shaping the warm‐ and cool‐water species' behavioral responses in a future ocean.

We show that ocean warming can restructure dominance hierarchies between warm‐ and cool‐water fish species, leading to a reversal in foraging dominance within tropicalizing marine ecosystems. While competitive reversals have been increasingly observed in terrestrial systems, where poleward‐shifting species often displace resident species under rising temperatures (Alexander et al., 2015; Louthan et al., 2015; Urban et al., 2012), the behavioral mechanisms mediating competitive reversal under ocean warming remain largely unexplored in our oceans. In insects and mammals, warming can alter foraging behavior, shift interspecific dominance, and promote the persistence of generalist species at their cold‐range edges (Sirén et al., 2021; Taulman & Lynn, 2014; Urban et al., 2012), while in plants, warming can restructure competitive hierarchies and facilitate the poleward expansion of stress‐tolerant or fast‐growing species (Alexander et al., 2015). We show that these patterns extend to marine systems, with warming eroding the competitive dominance of cool‐water reef fishes while enhancing the foraging performance of a warm‐water species in tropicalizing ecosystems in both the northern and southern hemispheres. If sustained, these changes in foraging performance may foreshadow a broader community transition in which warm‐affiliated species increasingly dominate ecological interactions, resource use, and community structure on temperate reefs (Soler et al., 2022). Thus, our findings support growing evidence that climate‐driven species redistributions will be accompanied by fundamental rewiring of ecological networks, reshaping both the composition and functioning of ecosystems across diverse biomes in the near future.

## CONCLUSIONS

A range‐extending tropical fish's foraging performance currently appears restricted by thermally unsuitable conditions and superior cool‐water competitors at their leading edges in temperate reef ecosystems. However, behavioral interference by cool‐water competitors during the early stages of tropicalization in temperate reefs may be weakened under future ocean warming through a reversal of competitive advantages, and this reversal was still observed irrespective of extreme pH reduction. In Japan, a strong marine heatwave had no detectable effects on the foraging performance of warm‐ and cool‐water fishes at a temperate reef. We suggest that chronic ocean warming rather than acute marine heatwaves is likely to mediate more significant shifts in warm‐ and cool‐water species foraging interactions on temperate reefs. Therefore, we conclude that under continuing ocean warming, even combined with ocean acidification, foraging dynamics will likely favor warm‐water fish over cool‐water competitors in temperate reefs, ultimately accelerating the tropicalization of temperate fish assemblages in the near future.

## FUNDING INFORMATION

This study was financially supported by a Discovery Projects grant from the Australian Research Council to Ivan Nagelkerken, Sean D. Connell, David J. Booth, and Timothy Ravasi (grant no. DP230101932), and the Okinawa Institute of Science and Technology, Kick‐start grant to Timothy Ravasi and Ivan Nagelkerken. This project contributes toward the International CO_2_ Natural Analogues (ICONA) Network and fieldwork conducted in Japan was partially funded by the Japan Society for the Promotion of Science (JSPS) Core‐to‐Core Program (grant number: JPJSCCA20210006). Ben P. Harvey was supported by JSPS KAKENHI (grant number: 23K26924).

## CONFLICT OF INTEREST STATEMENT

The authors declare no conflicts of interest.

## Supporting information


Appendix S1.

## Data Availability

Data (Mitchell, Nagelkerken, et al., 2025) are available in Figshare: https://doi.org/10.25909/28385819.v1.

## References

[ecy70226-bib-0001] Agostini, S. , B. P. Harvey , M. Milazzo , S. Wada , K. Kon , N. Floc'h , K. Komatsu , M. Kuroyama , and J. M. Hall‐Spencer . 2021. “Simplification, Not “Tropicalization”, of Temperate Marine Ecosystems under Ocean Warming and Acidification.” Global Change Biology 27(19): 4771–4784. 34268836 10.1111/gcb.15749

[ecy70226-bib-0002] Agostini, S. , B. P. Harvey , S. Wada , K. Kon , M. Milazzo , K. Inaba , and J. M. Hall‐Spencer . 2018. “Ocean Acidification Drives Community Shifts towards Simplified Non‐calcified Habitats in a Subtropical−Temperate Transition Zone.” Scientific Reports 8(1): 11354. 30054497 10.1038/s41598-018-29251-7 PMC6063920

[ecy70226-bib-0003] Agostini, S. , S. Wada , K. Kon , A. Omori , H. Kohtsuka , H. Fujimura , Y. Tsuchiya , et al. 2015. “Geochemistry of Two Shallow CO_2_ Seeps in Shikine Island (Japan) and their Potential for Ocean Acidification Research.” Regional Studies in Marine Science 2: 45–53.

[ecy70226-bib-0004] Alexander, J. , J. Diez , and J. Levine . 2015. “Novel Competitors Shape Species' Responses to Climate Change.” Nature 525(7570): 515–518. 26374998 10.1038/nature14952

[ecy70226-bib-0005] Alfonso, S. , M. Gesto , and B. Sadoul . 2021. “Temperature Increase and Its Effects on Fish Stress Physiology in the Context of Global Warming.” Journal of Fish Biology 98(6): 1496–1508. 33111333 10.1111/jfb.14599

[ecy70226-bib-0006] Anderson, M. J. 2001. “A New Method for Non‐parametric Multivariate Analysis of Variance.” Austral Ecology 26: 32–46.

[ecy70226-bib-0007] Anderson, M. J. , R. N. Gorley , and K. R. Clarke . 2008. PERMANOVA + for PRIMER: Guide to Software and Statistical Methods. Plymouth: PRIMER‐E.

[ecy70226-bib-0008] Australian Institute of Marine Science (AIMS) . 2024. “Sea Water Temperature Logger Data at One Tree Island, Great Barrier Reef From 25 Mar 2013 to 06 Aug 2024.” Australian Institute of Marine Science (AIMS). Dataset. https://apps.aims.gov.au/metadata/view/DE9C53FB-8B4F-1D2B-E054-6805CAA9DBF0.

[ecy70226-bib-0009] Beck, H. J. , D. A. Feary , A. M. Fowler , E. M. Madin , and D. J. Booth . 2016. “Temperate Predators and Seasonal Water Temperatures Impact Feeding of a Range Expanding Tropical Fish.” Marine Biology 163: 1–14.

[ecy70226-bib-0010] Bernal, M. A. , C. Schunter , R. Lehmann , D. J. Lightfoot , B. J. Allan , H. D. Veilleux , J. L. Rummer , P. L. Munday , and T. Ravasi . 2020. “Species‐Specific Molecular Responses of Wild Coral Reef Fishes during a Marine Heatwave.” Science Advances 6(12): eaay3423. 32206711 10.1126/sciadv.aay3423 PMC7080449

[ecy70226-bib-0011] Booth, D. J. , N. Bond , and P. I. Macreadie . 2011. “Detecting Range Shifts among Australian Fishes in Response to Climate Change.” Marine and Freshwater Research 62(9): 1027–1042.

[ecy70226-bib-0012] Booth, D. J. , W. F. Figueira , M. A. Gregson , and G. Beretta . 2007. “Occurrence of Tropical Fishes in Temperate Southeastern Australia: Role of the East Australian Current.” Estuarine, Coastal and Shelf Science 72(1–2): 102–114.

[ecy70226-bib-0013] Cattano, C. , S. Agostini , B. P. Harvey , S. Wada , F. Quattrocchi , G. Turco , K. Inaba , J. M. Hall‐Spencer , and M. Milazzo . 2020. “Changes in Fish Communities Due to Benthic Habitat Shifts under Ocean Acidification Conditions.” Science of the Total Environment 725: 138501. 32298893 10.1016/j.scitotenv.2020.138501

[ecy70226-bib-0014] Cattano, C. , J. Claudet , P. Domenici , and M. Milazzo . 2018. “Living in a High CO_2_ World: A Global Meta‐Analysis Shows Multiple Trait‐Mediated Fish Responses to Ocean Acidification.” Ecological Monographs 88(3): 320–335.

[ecy70226-bib-0015] Coni, E. O. C. , D. J. Booth , C. Ferreira , and I. Nagelkerken . 2021. “Behavioural Generalism Could Facilitate Coexistence of Tropical and Temperate Fishes under Climate Change.” Journal of Animal Ecology 91(1): 86–100. 34606086 10.1111/1365-2656.13599

[ecy70226-bib-0016] Coni, E. O. C. , D. J. Booth , and I. Nagelkerken . 2021. “Novel Species Interactions and Environmental Conditions Reduce Foraging Competency at the Temperate Range Edge of a Range‐Extending Coral Reef Fish.” Coral Reefs 40(5): 1525–1536.

[ecy70226-bib-0017] Coni, E. O. C. , D. J. Booth , and I. Nagelkerken . 2022. “Coral‐Reef Fishes Can Become More Risk‐Averse at their Poleward Range Limits.” Proceedings of the Royal Society B: Biological Sciences 289(1971): 20212676. 10.1098/rspb.2021.2676 PMC8941391 35317673

[ecy70226-bib-0018] Coni, E. O. C. , I. Nagelkerken , C. Ferreira , S. D. Connell , and D. J. Booth . 2021. “Ocean Acidification May Slow the Pace of Tropicalisation of Temperate Fish Communities.” Nature Climate Change 11(3): 249–256.

[ecy70226-bib-0066] Dickson, A. , and F. Millero . 1987. “A Comparison of the Equilibrium Constants for the Dissociation of Carbonic Acid in Seawater Media.” Deep Sea Research Part A 34(10): 1733–1743.

[ecy70226-bib-0019] Djurichkovic, L. D. , J. M. Donelson , A. M. Fowler , D. A. Feary , and D. J. Booth . 2019. “The Effects of Water Temperature on the Juvenile Performance of Two Tropical Damselfishes Expatriating to Temperate Reefs.” Scientific Reports 9(1): 13937. 31558794 10.1038/s41598-019-50303-z PMC6763422

[ecy70226-bib-0020] Donelson, J. M. , J. M. Sunday , W. F. Figueira , J. D. Gaitán‐Espitia , A. J. Hobday , C. R. Johnson , J. M. Leis , et al. 2019. “Understanding Interactions between Plasticity, Adaptation and Range Shifts in Response to Marine Environmental Change.” Philosophical Transactions of the Royal Society, B: Biological Sciences 374(1768): 20180186. 10.1098/rstb.2018.0186 PMC6365866 30966966

[ecy70226-bib-0021] Doney, S. C. , V. J. Fabry , R. A. Feely , and J. A. Kleypas . 2009. “Ocean Acidification: The Other CO_2_ Problem.” Annual Review of Marine Science 1: 169–192. 10.1146/annurev.marine.010908.163834 21141034

[ecy70226-bib-0022] Feary, D. A. , M. S. Pratchett , M. J. Emslie , A. Fowler , W. F. Figueira , O. J. Luiz , Y. Nakamura , and D. J. Booth . 2014. “Latitudinal Shifts in Coral Reef Fishes: Why some Species Do and Others Do Not Shift.” Fish and Fisheries 15(4): 593–615.

[ecy70226-bib-0023] Figueira, W. F. , P. Biro , D. J. Booth , and V. C. Valenzuela Davie . 2009. “Performance of Tropical Fish Recruiting to Temperate Habitats: Role of Ambient Temperature and Implications of Climate Change.” Marine Ecology Progress Series 384: 231–239.

[ecy70226-bib-0024] Figueira, W. F. , and D. J. Booth . 2010. “Increasing Ocean Temperatures Allow Tropical Fishes to Survive Overwinter in Temperate Waters.” Global Change Biology 16(2): 506–516.

[ecy70226-bib-0025] Froese, R. 2020. “Pers. Comm. R code (PrefTempBatch_5.R) to Estimate Preferred Temperature from Aquamaps (ver. 10/2019).” https://www.fishbase.se/summary/Abudefduf_vaigiensis.html.

[ecy70226-bib-0026] Frölicher, T. L. , E. M. Fischer , and N. Gruber . 2018. “Marine Heatwaves under Global Warming.” Nature 560(7718): 360–364. 30111788 10.1038/s41586-018-0383-9

[ecy70226-bib-0027] García Molinos, J. , H. L. Hunt , M. E. Green , C. Champion , J. R. Hartog , and G. T. Pecl . 2022. “Climate, Currents and Species Traits Contribute to Early Stages of Marine Species Redistribution.” Communications Biology 5(1): 1329. 36463333 10.1038/s42003-022-04273-0 PMC9719494

[ecy70226-bib-0028] Harvey, B. P. , R. Allen , S. Agostini , L. J. Hoffmann , K. Kon , T. C. Summerfield , S. Wada , and J. M. Hall‐Spencer . 2021. “Feedback Mechanisms Stabilise Degraded Turf Algal Systems at a CO_2_ Seep Site.” Communications Biology 4(1): 219. 33594188 10.1038/s42003-021-01712-2 PMC7901039

[ecy70226-bib-0029] Harvey, B. P. , K. Kon , S. Agostini , S. Wada , and J. M. Hall‐Spencer . 2021. “Ocean Acidification Locks Algal Communities in a Species‐Poor Early Successional Stage.” Global Change Biology 27(10): 2174–2187. 33423359 10.1111/gcb.15455

[ecy70226-bib-0030] Hayes, C. , A. Mitchell , R. Huerlimann , J. Jolly , C. Li , D. J. Booth , T. Ravasi , and I. Nagelkerken . 2025. “Stomach Microbiome Simplification of a Coral Reef Fish at Its Novel Cold‐Range Edge under Climate Change.” Molecular Ecology 34(7): e17704. 39985278 10.1111/mec.17704 PMC11934084

[ecy70226-bib-0031] Hayes, C. , A. Mitchell , C. Mellin , D. J. Booth , T. Ravasi , and I. Nagelkerken . 2024. “Ecological Generalism and Physiology Mediate Fish Biogeographic Ranges under Ocean Warming.” Proceedings of the Royal Society B: Biological Sciences 291(2015): 20232206. 10.1098/rspb.2023.2206 PMC10827425 38290546

[ecy70226-bib-0032] Hobday, A. J. , L. V. Alexander , S. E. Perkins , D. A. Smale , S. C. Straub , E. C. J. Oliver , J. A. Benthuysen , et al. 2016. “A Hierarchical Approach to Defining Marine Heatwaves.” Progress in Oceanography 141: 227–238.

[ecy70226-bib-0033] Kumagai, N. H. , J. García Molinos , H. Yamano , S. Takao , M. Fujii , and Y. Yamanaka . 2018. “Ocean Currents and Herbivory Drive Macroalgae‐to‐Coral Community Shift under Climate Warming.” Proceedings of the National Academy of Sciences 115(36): 8990–8995. 10.1073/pnas.1716826115 PMC6130349 30126981

[ecy70226-bib-0034] Louthan, A. M. , D. F. Doak , and A. L. Angert . 2015. “Where and when Do Species Interactions Set Range Limits?” Trends in Ecology & Evolution 30(12): 780–792. 26525430 10.1016/j.tree.2015.09.011

[ecy70226-bib-0065] Mehrbach, C. , C. Culberson , J. Hawley , and R. Pytkowicx . 1973. “Measurement of the Apparent Dissociation Constants of Carbonic Acid in Seawater at Atmospheric Pressure.” Limnology and Oceanography 18(6): 897–907.

[ecy70226-bib-0035] Milazzo, M. , S. Mirto , P. Domenici , and M. Gristina . 2013. “Climate Change Exacerbates Interspecific Interactions in Sympatric Coastal Fishes.” Journal of Animal Ecology 82(2): 468–477. 23039273 10.1111/j.1365-2656.2012.02034.x

[ecy70226-bib-0036] Mitchell, A. , D. J. Booth , and I. Nagelkerken . 2022. “Ocean Warming and Acidification Degrade Shoaling Performance and Lateralisation of Novel Tropical–Temperate Fish Shoals.” Global Change Biology 28(4): 1388–1401. 34918444 10.1111/gcb.16022

[ecy70226-bib-0037] Mitchell, A. , C. Hayes , D. J. Booth , and I. Nagelkerken . 2023a. “Projected Ocean Acidification and Seasonal Temperature Alter the Behaviour and Growth of a Range Extending Tropical Fish.” Coral Reefs 42(4): 919–929.

[ecy70226-bib-0038] Mitchell, A. , C. Hayes , D. J. Booth , and I. Nagelkerken . 2023b. “Ocean Acidification and Seasonal Water Temperatures Alter the Physiology of Competing Temperate and Coral Reef Fishes.” Science of the Total Environment 883: 163684. 37100135 10.1016/j.scitotenv.2023.163684

[ecy70226-bib-0039] Mitchell, A. , C. Hayes , E. O. C. Coni , D. J. Booth , and I. Nagelkerken . 2025. “Tropical Fishes Can Benefit More from Novel than Familiar Species Interactions at their Cold‐Range Edges.” Journal of Animal Ecology 94: 1997–2010. 40702600 10.1111/1365-2656.70100 PMC12484409

[ecy70226-bib-0040] Mitchell, A. , I. Nagelkerken , S. Connell , E. O. C. Coni , B. P. Harvey , S. Agostini , D. Booth , and T. Ravasi . 2025. “Range‐Extending Fish Become Competitive Dominants under Ocean Warming but Not Heatwaves or Acidification.” The University of Adelaide. Figshare. Dataset. 10.25909/28385819.v1. PMC12892380 41670047

[ecy70226-bib-0041] Nagelkerken, I. , and S. D. Connell . 2015. “Global Alteration of Ocean Ecosystem Functioning Due to Increasing Human CO_2_ Emissions.” Proceedings of the National Academy of Sciences 112(43): 13272–13277. 10.1073/pnas.1510856112 PMC4629388 26460052

[ecy70226-bib-0042] Paijmans, K. C. , D. J. Booth , and M. Y. Wong . 2020. “Predation Avoidance and Foraging Efficiency Contribute to Mixed‐Species Shoaling by Tropical and Temperate Fishes.” Journal of Fish Biology 96(3): 806–814. 32031243 10.1111/jfb.14277

[ecy70226-bib-0043] Parmesan, C. , and G. Yohe . 2003. “A Globally Coherent Fingerprint of Climate Change Impacts across Natural Systems.” Nature 421(6918): 37–42. 12511946 10.1038/nature01286

[ecy70226-bib-0044] Pecl, G. , M. Araújo , J. Bell , J. Blanchard , T. Bonebrake , I. Chen , T. Clark , et al. 2017. “Biodiversity Redistribution under Climate Change: Impacts on Ecosystems and Human Well‐Being.” Science 355(6332): eaii9214. 10.1126/science.aai9214 28360268

[ecy70226-bib-0045] Petit‐Marty, N. , I. Nagelkerken , S. D. Connell , and C. Schunter . 2021. “Natural CO_2_ Seeps Reveal Adaptive Potential to Ocean Acidification in Fish.” Evolutionary Applications 14(7): 1794–1806. 34295364 10.1111/eva.13239 PMC8288007

[ecy70226-bib-0064] Pierrot, D. , E. Lewis , and D. W. R. Wallace . 2006. “MS Excel Program Developed for CO2 System Calculations.” ORNL/CDIAC‐105a. Oak Ridge, TN: Carbon Dioxide Information Analysis Center. OakRidge National Laboratory; US Department of Energy.

[ecy70226-bib-0046] Pinsky, M. L. , B. Worm , M. J. Fogarty , J. L. Sarmiento , and S. A. Levin . 2013. “Marine Taxa Track Local Climate Velocities.” Science 341(6151): 1239–1242. 24031017 10.1126/science.1239352

[ecy70226-bib-0047] R Core Team . 2024. R: A Language and Environment for Statistical Computing. Vienna: R Foundation for Statistical Computing.

[ecy70226-bib-0048] Rodriguez‐Dominguez, A. , S. D. Connell , E. O. Coni , M. Sasaki , D. J. Booth , and I. Nagelkerken . 2022. “Phenotypic Responses in Fish Behaviour Narrow as Climate Ramps Up.” Climatic Change 171(1): 19.

[ecy70226-bib-0049] Sasaki, M. , A. Mitchell , D. J. Booth , and I. Nagelkerken . 2024. “Novel Ecological Interactions Alter Physiological Responses of Range‐Extending Tropical and Local Temperate Fishes under Ocean Warming.” Science of the Total Environment 913: 169413. 38114039 10.1016/j.scitotenv.2023.169413

[ecy70226-bib-0050] Sirén, A. P. , C. S. Sutherland , C. A. Bernier , K. J. Royar , J. R. Kilborn , C. B. Callahan , R. M. Cliché , L. S. Prout , and T. L. Morelli . 2021. “Abiotic Stress and Biotic Factors Mediate Range Dynamics on Opposing Edges.” Journal of Biogeography 48(7): 1758–1772.

[ecy70226-bib-0051] Smale, D. A. , and T. Wernberg . 2013. “Extreme Climatic Event Drives Range Contraction of a Habitat‐Forming Species.” Proceedings of the Royal Society B: Biological Sciences 280(1754): 20122829. 10.1098/rspb.2012.2829 PMC3574333 23325774

[ecy70226-bib-0052] Smale, D. A. , T. Wernberg , E. C. J. Oliver , M. Thomsen , B. P. Harvey , S. C. Straub , M. T. Burrows , et al. 2019. “Marine Heatwaves Threaten Global Biodiversity and the Provision of Ecosystem Services.” Nature Climate Change 9(4): 306–312.

[ecy70226-bib-0053] Smith, S. , R. Fox , D. J. Booth , and J. Donelson . 2018. “‘Stick with your Own Kind, or Hang with the Locals?’ Implications of Shoaling Strategy for Tropical Reef Fish on a Range‐Expansion Frontline.” Global Change Biology 24(4): 1663–1672. 29334689 10.1111/gcb.14016

[ecy70226-bib-0054] Soler, G. A. , G. J. Edgar , N. S. Barrett , R. D. Stuart‐Smith , E. Oh , A. Cooper , K. R. Ridgway , and S. D. Ling . 2022. “Warming Signals in Temperate Reef Communities Following More than a Decade of Ecological Stability.” Proceedings of the Royal Society B 289(1989): 20221649. 36515119 10.1098/rspb.2022.1649 PMC9748771

[ecy70226-bib-0055] Taulman, J. F. , and W. R. Lynn . 2014. “Range Expansion and Distributional Limits of the Nine‐Banded Armadillo in the United States: An Update of Taulman & Robbins (1996).” Journal of Biogeography 41(8): 1626–1630.

[ecy70226-bib-0056] Tea, Y. K. , and A. C. Gill . 2020. “Systematic Reappraisal of the Anti‐Equatorial Fish Genus *Microcanthus* Swainson (Teleostei: Microcanthidae), with Redescription and Resurrection of *Microcanthus joyceae* Whitley.” Zootaxa 4802(1): 41–60. 10.11646/zootaxa.4802.1.3 33056631

[ecy70226-bib-0057] Tuomainen, U. , and U. Candolin . 2011. “Behavioural Responses to Human‐Induced Environmental Change.” Biological Reviews 86(3): 640–657. 20977599 10.1111/j.1469-185X.2010.00164.x

[ecy70226-bib-0058] Twiname, S. , Q. P. Fitzgibbon , A. J. Hobday , C. G. Carter , M. Oellermann , and G. T. Pecl . 2022. “Resident Lobsters Dominate Food Competition with Range‐Shifting Lobsters in an Ocean Warming Hotspot.” Marine Ecology Progress Series 685: 171–181.

[ecy70226-bib-0059] Urban, M. C. , J. J. Tewksbury , and K. S. Sheldon . 2012. “On a Collision Course: Competition and Dispersal Differences Create no‐Analogue Communities and Cause Extinctions during Climate Change.” Proceedings of the Royal Society B: Biological Sciences 279: 2072–2080. 10.1098/rspb.2011.2367 PMC3311897 22217718

[ecy70226-bib-0060] Van Wert, J. C. , K. Birnie‐Gauvin , J. Gallagher , E. A. Hardison , K. Landfield , D. E. Burkepile , and E. J. Eliason . 2024. “Despite Plasticity, Heatwaves Are Costly for a Coral Reef Fish.” Scientific Reports 14(1): 13320. 38858427 10.1038/s41598-024-63273-8 PMC11164959

[ecy70226-bib-0061] Vergés, A. , P. D. Steinberg , M. E. Hay , A. G. B. Poore , A. H. Campbell , E. Ballesteros , K. L. Heck , et al. 2014. “The Tropicalisation of Temperate Marine Ecosystems: Climate‐Mediated Changes in Herbivory and Community Phase Shifts.” Proceedings of the Royal Society B: Biological Sciences 281(1789): 20140846. 10.1098/rspb.2014.0846 PMC4100510 25009065

[ecy70226-bib-0062] Wickham, H. 2016. ggplot2: Elegant Graphics for Data Analysis. New York: Springer‐Verlag New York.

[ecy70226-bib-0063] Wu, L. , W. Cai , L. Zhang , H. Nakamura , A. Timmermann , T. Joyce , M. J. McPhaden , et al. 2012. “Enhanced Warming over the Global Subtropical Western Boundary Currents.” Nature Climate Change 2(3): 161–166.

