# Supplementary material for: Range‐extending fish become competitive dominants under ocean warming but not heatwaves or acidification
Source: Ecology. 2026 Feb 11;107(2):e70226. doi: 10.1002/ecy.70226 (PMC12892380; doi:10.1002/ecy.70226)
Supplement: Supplementary file 1 — Appendix S1. [file ECY-107-e70226-s001.pdf]

Appendix S1

Range-extending fish become competitive dominants under ocean warming but not heat waves or acidification

Angus Mitchell, Ericka O.C. Coni, Sean D. Connell, David J. Booth, Ben P. Harvey, Sylvain Agostini, Timothy Ravasi, Ivan Nagelkerken

Journal: ***Ecology***

Table S1: Sample sizes (n) behavioural observations collected for juvenile warm-water (*Abudefduf vaigiensis*) and cool-water fishes (Australia: *Microcanthus joyceae*; Japan: *Microcanthus strigatus*) across reefs in Australia and Japan. Sample sizes for the temperate reef in Japan is also presented for before and during the strong marine heatwave event.

**Latitudinal Gradient in Australia**		
**Reefs**	***Abudefduf vaigiensis***	***Microcanthus joyceae***
Tropical	9	9
Subtropical	15	20
Warm temperate	23	20
Cold temperate	21	15
**Natural analogues in Japan**		
**Reef**	***Abudefduf vaigiensis***	***Microcanthus joyceae***
Temperate	15	14
Tropicalized	6	9
Extreme	9	4
**Marine heatwave at the temperate reef in Japan**		
**Marine Heatwave**	***Abudefduf vaigiensis***	***Microcanthus joyceae***
Before	8	7
During	7	7

Table S2: Ethogram of the behavioural traits used as a proxy of competitive ability between tropical and temperate fish species in *in situ* experiments. All behaviours were observed continuously within a recording across the sampled reefs in Australia and Japan.

**Behavioural traits**	**Description**
Bite rate	total number of bites taken at the released prey during the recording
Prey inspection rate	total number of times that the individual approached the tube (point of prey release) within 5 body lengths after release of the prey
Chasing rate	total number of times that an individual swam aggressively towards another individual (either a conspecific, or a tropical or temperate heterospecific)
Escaping rate	total number of times that an individual fled from an aggressor (conspecific, or tropical or temperate heterospecific)
Prey approach time	amount of time for an individual to approach the prey after it was first released (swimming towards the prey)
Minimum distance to prey	closest distance (cm) that the individual approached the tube during the entire observation time
Retreat rate	total number of times that an individual attempted to approach the prey (minimum distance of ≤ 5 body lengths) but decided to abruptly turn around and retreat to or close to its previous position

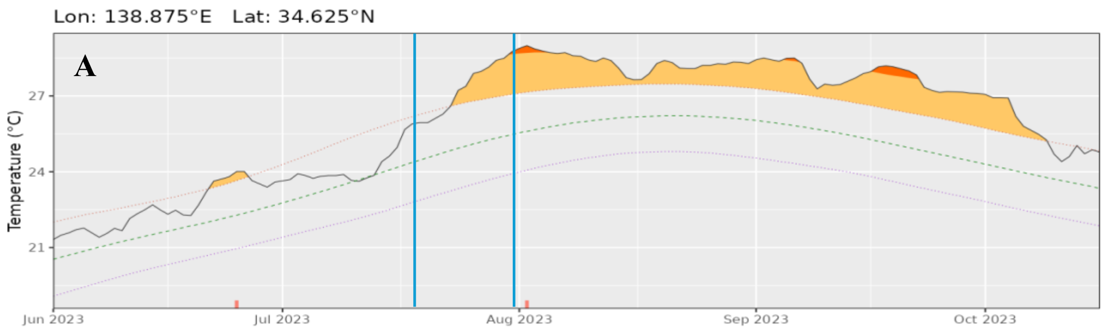

	Event	Lon	Lat	#	Category	Duration	Start Date	Peak Date	End Date	Mean Intensity	Max. Intensity	Cum. Intensity
1	MHW	138.875	34.625	137	I Moderate	5	2023-06-22	2023-06-25	2023-06-26	1.622	1.73	8.11
2	MHW	138.875	34.625	138	II Strong	78	2023-07-24	2023-08-02	2023-10-09	2.426	3.39	189.24

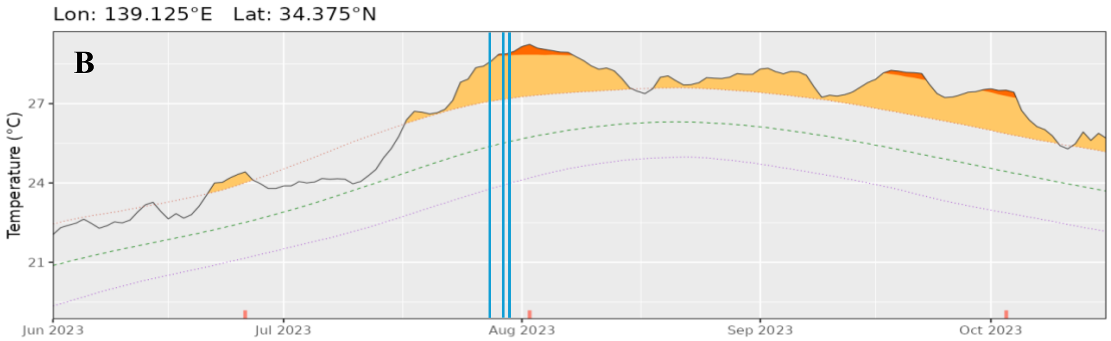

	Event	Lon	Lat	#	Category	Duration	Start Date	Peak Date	End Date	Mean Intensity	Max. Intensity	Cum. Intensity
1	MHW	139.125	34.375	141	I Moderate	5	2023-06-22	2023-06-26	2023-06-26	1.82	1.91	9.1
2	MHW	139.125	34.375	142	II Strong	30	2023-07-17	2023-08-02	2023-08-15	2.629	3.51	78.87

Figure S1: Visualisation of unprecedented marine heatwave for (A): temperate and (B): tropicalized and extreme reefs between 06/06/2023–19/10/2023. Blue lines denote behaviour sampling dates at the Temperate reef ((A) before heatwave: 17th of July, 2023; and during heatwave: 30th of July, 2023) and at the Tropicalized and Extreme reefs (25th and 27th of July, 2023 (B)). Marine heatwave data and visualisation was extracted from marineheatwavetracker.com.org

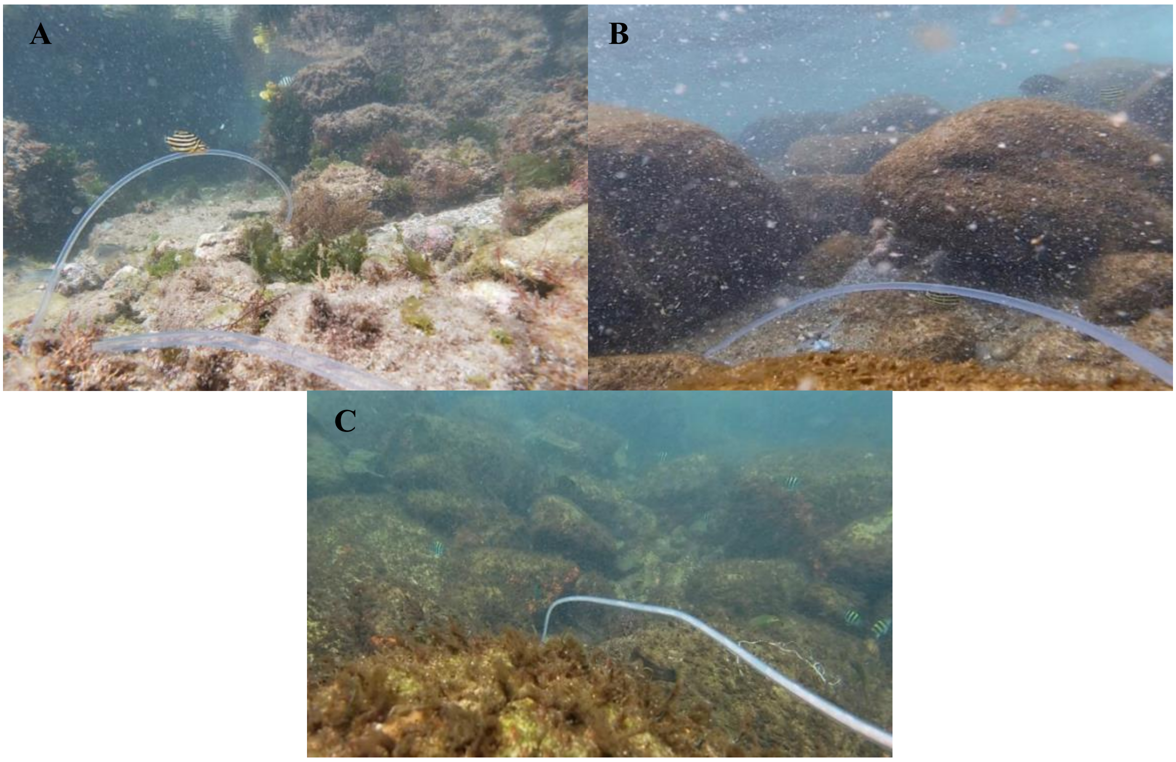

Figure S2: Seascapes of near-tidal natural analogue reefs representative of temperate (during summer) (A), tropicalized (B), and extreme (C) reef localities sampled in Japan. All photographs (panels A–C) were taken by Angus Mitchell, an author of this manuscript, and are original content.

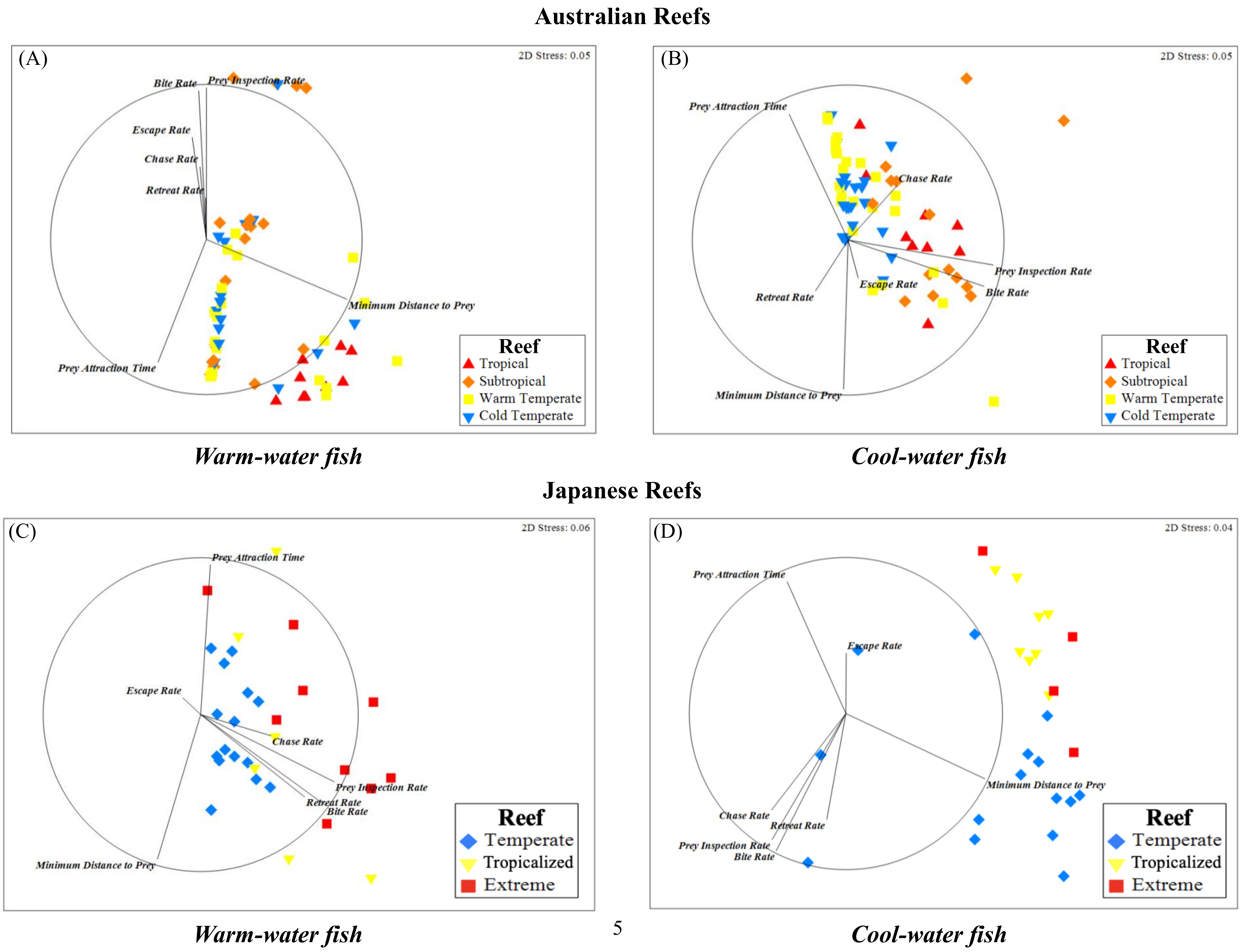
Australian Reefs
(A)
2D Stress: 0.05
Bite Rate
Prey Inspection Rate
Escape Rate
Chase Rate
Retreat Rate
Minimum Distance to Prey
Prey Attraction Time
Reef
Tropical
Subtropical
Warm Temperate
Cold Temperate
Warm-water fish
(B)
2D Stress: 0.05
Prey Attraction Time
Chase Rate
Prey Inspection Rate
Bite Rate
Escape Rate
Retreat Rate
Minimum Distance to Prey
Reef
Tropical
Subtropical
Warm Temperate
Cold Temperate
Cool-water fish
Japanese Reefs
(C)
2D Stress: 0.06
Prey Attraction Time
Escape Rate
Chase Rate
Prey Inspection Rate
Retreat Rate
Bite Rate
Minimum Distance to Prey
Reef
Temperate
Tropicalized
Extreme
Warm-water fish
(D)
2D Stress: 0.04
Prey Attraction Time
Escape Rate
Minimum Distance to Prey
Chase Rate
Retreat Rate
Prey Inspection Rate
Bite Rate
Reef
Temperate
Tropicalized
Extreme
Cool-water fish

Figure S3: Non-metric multidimensional scaling (nMDS) ordination plots showing variation in multivariate foraging behaviour of warm-water and cool-water fish species across reef types in Australia (A–B) and Japan (C–D). Each point represents a single individual, with position determined by seven log(X+1)-transformed behavioural metrics. Vectors indicate the direction and strength of each behavioural variable's correlation with the ordination space. (A) Warm-water species across tropical (red triangles), subtropical (orange diamonds), warm-temperate (yellow squares), and cold-temperate (blue inverted triangles) reefs in Australia. (B) Cool-water species across the same Australian reefs. (C) Warm-water species across Japanese temperate (red squares), tropicalized (yellow inverted triangles), and extreme (blue diamonds) reefs. (D) Cool-water species across the same Japanese reef types. Stress values for 2D solutions are shown in the top right of each panel.

Fish foraging behaviour in Australia

Table S3: MANOVA output of foraging behaviours of warm-water and cool-water fish species in Australia and resulting pairwise tests. Note: **bold** P-values denote significant differences (P < 0.05). ndf = numerator degrees of freedom, SS = sum of squares, MS = mean square, ddf = denominator degrees of freedom.

Factor	ndf	SS	MS	ddf	F-value	P-value
**Reef**	3	8678.3	2892.8	124	6.651	**<0.001**
**Species**	1	13078	13078	124	30.07	**<0.001**
**Reeef×Species**	3	6311.1	2103.7	124	4.8369	**<0.001**
**Residuals**	124	53931	434.93			
**Total**	131	90367				

Pairwise tests for levels of factor 'Species' within level 'Tropical' of factor 'Reef'

Comparison	ddf	t-value	P-value
Abudefduf vaigiensis, Microcanthus joyceae	16	2.297	**0.025**

Pairwise tests for levels of factor 'Species' within level 'Subtropical' of factor 'Reef'

Comparison	ddf	t-value	P-value
Abudefduf vaigiensis, Microcanthus joyceae	33	3.371	**<0.001**

Pairwise tests for levels of factor 'Species' within level 'Warm Temperate' of factor 'Reef'

Comparison	ddf	t-value	P-value
Abudefduf vaigiensis, Microcanthus joyceae	41	3.587	**<0.001**

Pairwise tests for levels of factor 'Species' within level 'Cold Temperate' of factor 'Reef'

Comparison	ddf	t-value	P-value
Abudefduf vaigiensis, Microcanthus joyceae	34	6.028	**<0.001**

Term 'Species×Region" for pairs of levels of factor 'Reef' within level '*Abudefduf vaigiensis'* of factor 'Species'

Comparison	ddf	t-value	P-value
**Tropical, Cold Temperate**	28	3.177	**0.002**
**Tropical, Warm Temperate**	30	1.727	0.093
**Tropical, Subtropical**	22	0.640	0.688
**Cold Temperate, Warm Temperate**	42	Negative	–
**Cold Temperate, Subtropical**	34	3.860	**<0.001**
**Warm Temperate, Subtropical**	36	2.474	**0.009**

Term 'Species×Region' for pairs of levels of factor 'Reef' within level '*Microcanthus joyceae*' of factor 'Species'

Comparison	ddf	t-value	P-value
**Tropical, Cold Temperate**	22	3.700	**<0.001**
**Tropical, Warm Temperate**	27	2.445	**0.007**
**Tropical, Subtropical**	27	3.563	**<0.001**
**Cold Temperate, Warm Temperate**	33	1.666	0.075
**Cold Temperate, Subtropical**	33	0.828	0.424
**Tropical, Cold Temperate**	38	2.457	**0.014**

Fish foraging behaviours in Japan

Table S4: MANOVA output of foraging behaviours of warm-water and cool-water fish species in Japan and resulting pairwise tests. Note: **bold** P-values denote significant differences (P < 0.05). ndf = numerator degrees of freedom, SS = sum of squares, MS = mean square, ddf = denominator degrees of freedom.

Factor	ndf	SS	MS	ddf	F-value	P-value
**Species**	1	891.09	891.09	51	4.817	**0.012**
**Reef**	2	1086.8	543.41	51	2.937	**0.030**
**Species×Reef**	2	3218.3	1609.1	51	8.698	**<0.001**
**Residuals**	51	9435.3	185.01			
**Total**	56	14843				

**Pairwise tests for levels of factor ‘Species’ within level ‘Temperate’ of factor ‘Reef’**			
**Comparison**	**ddf**	**t-value**	**P-value**
Abudefduf vaigiensis, Microcanthus strigatus	28	3.473	**<0.001**
**Pairwise tests for levels of factor ‘Species’ within level ‘Tropicalized of factor ‘Reef’**			
**Comparison**	**ddf**	**t-value**	**P-value**
Abudefduf vaigiensis, Microcanthus strigatus	12	2.740	**0.016**
**Pairwise tests for levels of factor ‘Species’ within level ‘Extreme’ of factor ‘Reef’**			
**Comparison**	**ddf**	**t-value**	**P-value**
Abudefduf vaigiensis, Microcanthus strigatus	11	1.861	0.071
**Term ‘Species×Reef” for pairs of levels of factor 'Reef' within level ‘*Abudefduf vaigiensis’* of factor ‘Species’**			
**Comparison**	**ddf**	**t-value**	**P-value**
**Temperate, Tropicalized**	19	0.990	0.344
**Temperate, Extreme**	22	2.692	**0.002**
**Tropicalized, Extreme**	13	0.722	0.551
**Term ‘Species×Reef’ for pairs of levels of factor ‘Reef’ within level ‘*Microcanthus strigatus*’ of factor ‘Species’**			
**Comparison**		**t-value**	**P-value**
**Temperate, Tropicalized**	21	3.554	**0.001**
**Temperate, Extreme**	17	2.134	**0.018**
**Tropicalized, Extreme**	10	0.832	0.433

Table S5: SIMPER analysis showing the contribution of individual behaviours to the average dissimilarity in the warm-water fish's (*Abudefduf vaigiensis)* foraging performance between Australian reefs. Behaviours are ranked by their contribution (%) to total dissimilarity.

**Dissimilarity between tropical and subtropical reefs**						
**Behaviour**	**Group average value: Tropical**	**Group average value: Subtropical**	**Average Dissimilarity**	**Dissimilarity/ Standard Deviation**	**Contribution (%)**	**Cumulative Contribution (%)**
Minimum distance to prey	3.440	3.170	6.740	1.050	36.46	36.46
Prey attraction time	3.930	3.810	3.820	1.320	20.69	57.15
Bite rate	1.370	1.230	3.720	1.390	20.12	77.27
Prey inspection rate	0.650	0.970	3.380	1.130	18.32	95.59
Retreat rate	0.058	0.058	0.490	0.770	2.68	98.27
Escape rate	0.025	0.028	0.250	0.530	1.33	99.60
Chase rate	0.000	0.012	0.070	0.270	0.40	100.00
**Dissimilarity between cold temperate and warm temperate reefs**						
**Behaviour**	**Group average value: Cold temperate**	**Group average value: Warm temperate**	**Average Dissimilarity**	**Dissimilarity/ Standard Deviation**	**Contribution (%)**	**Cumulative Contribution (%)**
Minimum distance to prey	3.100	2.990	6.070	1.360	40.89	40.89
Prey attraction time	4.270	4.040	3.500	0.580	23.59	64.48
Bite rate	0.300	0.360	2.270	1.120	15.30	79.78
Prey inspection rate	0.120	0.292	1.640	0.900	11.08	90.86
Retreat rate	0.122	0.102	0.920	1.140	6.19	97.05
Escape rate	0.382	0.056	0.440	0.800	2.95	100.00
Chase rate	0.000	0.000	0.080	Undefined	0.00	100.00
**Dissimilarity between cold temperate and subtropical reefs**						
**Behaviour**	**Group average value: Cold temperate**	**Group average value: Subtropical**	**Average Dissimilarity**	**Dissimilarity/ Standard Deviation**	**Contribution (%)**	**Cumulative Contribution (%)**
Minimum distance to prey	3.100	3.170	7.210	1.160	32.18	32.18
Bite rate	0.300	1.230	5.510	1.630	24.59	56.78
Prey inspection rate	0.120	0.970	4.880	1.410	21.78	78.56
Prey attraction time	4.270	3.810	3.670	1.160	16.40	94.96
Retreat rate	0.122	0.058	0.720	1.310	3.22	98.18
Escape rate	0.382	0.028	0.330	0.720	1.45	99.64
Chaste rate	0.000	0.010	0.080	0.270	0.36	100.00
**Dissimilarity between warm temperate and subtropical reefs**						
**Behaviour**	**Group average value: Warm temperate**	**Group average value: Subtropical**	**Average Dissimilarity**	**Dissimilarity/ Standard Deviation**	**Contribution (%)**	**Cumulative Contribution (%)**
Minimum distance to prey	2.990	3.170	8.010	1.250	33.35	33.35
Prey attraction time	4.040	3.810	5.530	1.580	23.04	56.39
Bite rate	0.360	1.230	4.850	0.880	20.21	76.60
Prey inspection rate	0.292	0.970	4.410	1.250	18.37	94.97

Dissimilarity between warm temperate and subtropical reefs						
Retreat rate	0.102	0.058	0.720	0.800	3.01	97.98
Escape rate	0.056	0.028	0.400	0.710	1.67	99.66
Chase rate	0	0.012	0.080	0.270	0.34	100.00
**Dissimilarity between cold temperate and tropical reefs**						
**Behaviour**	**Group average value: Cold temperate**	**Group average value: Tropical**	**Average Dissimilarity**	**Dissimilarity/ Standard Deviation**	**Contribution (%)**	**Cumulative Contribution (%)**
Bite rate	0.300	1.370	6.160	1.940	33.37	33.37
Minimum distance to prey	3.100	3.440	5.250	1.240	28.48	61.85
Prey attraction time	4.270	3.930	3.080	1.300	16.71	78.56
Prey inspection rate	0.120	0.650	2.920	0.990	15.83	94.39
Retreat rate	0.122	0.058	0.730	1.290	3.96	98.35
Escape rate	0.038	0.025	0.300	0.700	1.65	100.00
Chase rate	0.000	0.000	0.000	Undefined	0.00	100.00
**Dissimilarity between warm temperate and tropical reefs**						
**Behaviour**	**Group average value: Warm temperate**	**Group average value: Tropical**	**Average Dissimilarity**	**Dissimilarity/ Standard Deviation**	**Contribution (%)**	**Cumulative Contribution (%)**
Minimum distance to prey	2.990	3.440	6.370	1.38	30.36	30.36
Bite rate	0.360	1.370	6.120	1.81	29.16	59.52
Prey attraction time	4.040	3.930	4.340	0.80	20.69	80.20
Prey inspection rate	0.292	0.650	3.060	1.32	14.55	94.76
Retreat rate	0.102	0.058	0.720	0.78	3.43	98.19
Escape rate	0.056	0.025	0.380	0.69	1.81	100.00
Chaste rate	0.000	0.000	0.000	Undefined	0.00	100.00

Table S6: SIMPER analysis showing the contribution of individual behaviours to the average dissimilarity in the cool-water fish's (*Microcanthus joyceae*) foraging performance between Australian reefs. Behaviours are ranked by their contribution (%) to total dissimilarity.

**Dissimilarity between tropical and subtropical reefs**						
**Behaviour**	**Group average value: Tropical**	**Group average value: Subtropical**	**Average Dissimilarity**	**Dissimilarity/ Standard Deviation**	**Contribution (%)**	**Cumulative Contribution (%)**
Minimum distance to prey	2.92	0.182	17.230	3.070	37.25	37.25
Bite rate	0.459	2.210	10.860	1.280	23.48	60.72
Prey attraction time	4.320	3.550	7.700	1.190	16.63	77.35
Prey inspection rate	0.233	1.490	6.440	0.650	13.91	91.27
Escape rate	0.000	0.311	1.680	0.670	3.63	94.89
Chase rate	0.011	0.219	1.230	0.660	2.65	97.55
Retreat rate	0.101	0.131	1.140	0.650	2.45	100.00
**Dissimilarity between cold temperate and warm temperate reefs**						
**Behaviour**	**Group average value: Cold temperate**	**Group average value: Warm temperate**	**Average Dissimilarity**	**Dissimilarity/ Standard Deviation**	**Contribution (%)**	**Cumulative Contribution (%)**
Minimum distance to prey	0.594	1.230	8.450	0.860	28.22	28.22
Bite rate	1.830	1.220	8.090	1.360	27.02	55.24
Prey attraction time	4.120	4.330	5.590	1.220	18.68	73.92
Prey inspection rate	1.230	0.727	3.470	0.510	11.59	85.52
Retreat rate	0.202	0.263	1.930	0.910	6.45	91.97
Escape rate	0.314	0.139	1.930	0.930	6.44	98.40
Chase rate	0.077	0.010	0.480	0.410	1.60	100.00
**Dissimilarity between cold temperate and subtropical reefs**						
**Behaviour**	**Group average value: Cold temperate**	**Group average value: Subtropical**	**Average Dissimilarity**	**Dissimilarity/ Standard Deviation**	**Contribution (%)**	**Cumulative Contribution (%)**
Prey attraction time	4.120	3.550	9.840	1.420	28.73	28.73
Bite rate	1.830	2.210	7.360	0.670	21.49	50.23
Prey inspection rate	1.230	1.490	7.260	1.330	21.21	71.44
Minimum distance to prey	0.594	0.182	4.250	0.570	12.41	83.85
Escape rate	0.314	0.311	2.500	0.950	7.29	91.13
Retreat rate	0.202	0.131	1.620	0.660	4.73	95.87
Chaste rate	0.077	0.219	1.410	0.740	4.13	100.00
**Dissimilarity between warm temperate and subtropical reefs**						
**Behaviour**	**Group average value: Warm temperate**	**Group average value: Subtropical**	**Average Dissimilarity**	**Dissimilarity/ Standard Deviation**	**Contribution (%)**	**Cumulative Contribution (%)**
Bite rate	1.220	2.210	10.590	1.340	28.25	28.25
Minimum distance to prey	1.230	0.182	7.590	0.780	20.24	48.50
Prey attraction time	4.330	3.550	7.430	1.260	19.82	68.32
Prey inspection rate	0.727	1.490	6.760	0.650	18.02	86.34

**Dissimilarity between warm temperate and subtropical reefs**						
Escape rate	0.139	0.311	1.950	0.860	5.21	91.55
Retreat rate	0.263	0.131	1.920	0.890	5.12	96.66
Chase rate	0.010	0.219	1.250	0.650	3.34	100.00
**Dissimilarity between cold temperate and tropical reefs**						
**Behaviour**	**Group average value: Tropical**	**Group average value: Cold temperate**	**Average Dissimilarity**	**Dissimilarity/ Standard Deviation**	**Contribution (%)**	**Cumulative Contribution (%)**
Minimum distance to prey	2.920	0.594	15.560	2.450	42.29	42.29
Bite rate	0.459	1.830	8.450	1.380	22.96	65.24
Prey inspection rate	0.233	1.230	6.040	1.250	16.43	81.67
Prey attraction time	4.320	4.120	3.210	0.510	8.73	90.40
Retreat rate	0.101	0.314	1.760	0.750	4.78	95.18
Escape rate	0.000	0.202	1.300	0.720	3.55	98.72
Chase rate	0.111	0.077	0.470	0.420	1.28	100.00
**Dissimilarity between warm temperate and tropical reefs**						
**Behaviour**	**Group average value: Warm temperate**	**Group average value: Tropical**	**Average Dissimilarity**	**Dissimilarity/ Standard Deviation**	**Contribution (%)**	**Cumulative Contribution (%)**
Minimum distance to prey	1.230	2.920	14.32	1.780	50.64	50.64
Bite rate	1.220	0.459	5.910	1.180	20.88	71.53
Prey inspection rate	0.727	0.233	3.650	1.190	12.89	84.42
Prey attraction time	4.330	4.320	2.010	1.040	7.11	91.53
Retreat rate	0.263	0.101	1.440	0.910	5.10	96.62
Escape rate	0.139	0.000	0.830	0.810	2.93	99.55
Chaste rate	0.010	0.111	0.130	0.400	0.45	100.00

Table S7: SIMPER analysis showing the contribution of individual behaviours to the average dissimilarity in the warm-water fish's (*Abudefduf vaigiensis*) foraging performance between Japanese reefs. Behaviours are ranked by their contribution (%) to total dissimilarity.

**Dissimilarity between temperate and tropicalized reefs**						
**Behaviour**	**Group average value: Temperate**	**Group average value: Tropicalized**	**Average Dissimilarity**	**Dissimilarity/ Standard Deviation**	**Contribution (%)**	**Cumulative Contribution (%)**
Prey attraction time	3.670	3.530	4.030	1.520	25.60	25.60
Minimum distance to prey	4.010	3.670	3.970	0.870	25.10	50.80
Bite rate	0.900	1.390	3.850	1.490	24.44	75.24
Prey inspection rate	0.200	0.550	1.910	0.850	12.14	87.38
Escape rate	0.220	0.320	1.470	0.920	9.32	96.70
Chase rate	0.010	0.050	0.300	0.950	1.91	98.61
Retreat rate	0.040	0.010	0.220	0.820	1.39	100.00
**Dissimilarity between temperate and extreme reefs**						
**Behaviour**	**Group average value: Temperate**	**Group average value: Extreme**	**Average Dissimilarity**	**Dissimilarity/ Standard Deviation**	**Contribution (%)**	**Cumulative Contribution (%)**
Bite rate	0.900	1.720	5.140	1.730	31.91	31.91
Minimum distance to prey	4.010	3.550	3.150	1.270	19.53	19.53
Prey inspection rate	0.200	0.740	2.880	1.730	17.87	69.32
Prey attraction time	3.670	3.930	2.780	1.340	17.24	86.56
Retreat rate	0.220	0.410	1.440	1.600	8.96	95.52
Chase rate	0.010	0.090	0.440	0.670	2.73	98.25
Escape rate	0.040	0.030	0.280	0.860	1.75	100.00
**Dissimilarity between tropicalized and extreme reefs**						
**Behaviour**	**Group average value: Tropicalized**	**Group average value: Extreme**	**Average Dissimilarity**	**Dissimilarity/ Standard Deviation**	**Contribution (%)**	**Cumulative Contribution (%)**
Bite rate	1.390	1.720	4.610	1.430	25.28	25.28
Minimum distance to prey	3.670	3.550	4.340	1.280	23.79	49.07
Prey attraction time	3.530	3.930	3.940	1.390	21.64	70.71
Prey inspection rate	0.550	0.740	2.770	1.620	15.20	85.91
Retreat rate	0.320	0.410	1.900	1.460	10.44	96.35
Chase rate	0.050	0.090	0.500	0.860	2.74	99.09
Escape rate	0.010	0.030	0.170	0.520	0.91	100.00

Table S8: SIMPER analysis showing the contribution of individual behaviours to the average dissimilarity in the cool-water fish's (*Microcanthus strigatus*) foraging performance between Japanese reefs. Behaviours are ranked by their contribution (%) to total dissimilarity.

**Dissimilarity between temperate and tropicalized reefs**						
**Behaviour**	**Group average value: Temperate**	**Group average value: Tropicalized**	**Average Dissimilarity**	**Dissimilarity/ Standard Deviation**	**Contribution (%)**	**Cumulative Contribution (%)**
Bite rate	2.120	0.480	8.800	2.150	29.99	29.99
Minimum distance to prey	3.080	2.720	8.760	1.720	29.85	59.84
Prey attraction time	3.620	4.420	4.460	1.340	15.19	75.03
Prey inspection rate	0.970	0.160	4.260	1.460	14.53	89.56
Escape rate	0.450	0.160	1.770	1.080	6.04	95.60
Chase rate	0.250	0.010	1.260	0.900	4.30	99.90
Retreat rate	0.000	0.010	0.030	0.370	0.10	100.00
**Dissimilarity between temperate and extreme reefs**						
**Behaviour**	**Group average value: Temperate**	**Group average value: Extreme**	**Average Dissimilarity**	**Dissimilarity/ Standard Deviation**	**Contribution (%)**	**Cumulative Contribution (%)**
Bite rate	2.120	0.600	8.280	1.740	29.69	29.69
Minimum distance to prey	3.080	3.160	8.250	1.080	29.56	59.25
Prey inspection rate	0.970	0.180	4.160	1.400	14.90	74.15
Prey attraction time	3.620	4.120	3.950	1.260	14.16	88.31
Retreat rate	0.450	0.120	1.940	1.120	6.94	95.25
Chase rate	0.250	0.000	1.270	0.890	4.57	99.82
Escape rate	0.000	0.010	0.050	0.570	0.18	100.00
**Dissimilarity between tropicalized and extreme reefs**						
**Behaviour**	**Group average value: Tropicalized**	**Group average value: Extreme**	**Average Dissimilarity**	**Dissimilarity/ Standard Deviation**	**Contribution (%)**	**Cumulative Contribution (%)**
Minimum distance to prey	2.720	3.160	6.980	1.720	49.39	49.39
Bite rate	0.480	0.600	3.050	1.360	21.56	70.95
Prey attraction time	4.420	4.120	2.340	1.200	16.54	87.50
Prey inspection rate	0.160	0.180	0.840	1.430	5.95	93.45
Retreat rate	0.160	0.120	0.760	1.410	5.39	98.83
Chase rate	0.010	0.000	0.090	0.550	0.61	99.44
Escape rate	0.010	0.010	0.080	0.680	0.56	100.00

Prey inspection rates in Australia

Table S9: ANOVA output of prey inspection rates of warm-water and cool-water fish species in Australia and resulting pairwise tests. Note: **bold** P-values denote significant differences (P < 0.05). ndf = numerator degrees of freedom, SS = sum of squares, MS = mean square, ddf = denominator degrees of freedom.

Factor	ndf	SS	MS	ddf	F-value	P-value
**Reef**	3	0.070	0.023	124	9.118	**<0.001**
**Species**	1	0.023	0.023	124	9.109	**0.003**
**Reef×Species**	3	0.045	0.0149	124	5.814	**<0.001**
**Residuals**	124	0.318	0.003			
**Total**	131	0.496				

Pairwise tests for levels of factor 'Species' within level 'Tropical' of factor 'Reef'

Comparison	ddf	t-value	P-value
Abudefduf vaigiensis, Microcanthus joyceae	16	1.802	0.085

Pairwise tests for levels of factor 'Species' within level 'Subtropical' of factor 'Reef'

Comparison	ddf	t-value	P-value
Abudefduf vaigiensis, Microcanthus joyceae	33	1.044	0.310

Pairwise tests for levels of factor 'Species' within level 'Warm Temperate' of factor 'Reef'

Comparison	ddf	t-value	P-value
Abudefduf vaigiensis, Microcanthus joyceae	41	7.832	**<0.001**

Pairwise tests for levels of factor 'Species' within level 'Cold Temperate' of factor 'Reef'

Comparison	ddf	t-value	P-value
Abudefduf vaigiensis, Microcanthus joyceae	34	10.304	**<0.001**

Term 'Species×Region" for pairs of levels of factor 'Reef' within level '*Abudefduf vaigiensis*' of factor 'Species'

Comparison	ddf	t-value	P-value
**Tropical, Cold Temperate**	28	4.240	**<0.001**
**Tropical, Warm Temperate**	30	1.732	0.092
**Tropical, Subtropical**	22	1.155	0.253
**Cold Temperate, Warm Temperate**	42	2.702	**0.009**
**Cold Temperate, Subtropical**	34	6.709	**<0.001**
**Warm Temperate, Subtropical**	36	3.780	**<0.001**

Term 'Species×Region' for pairs of levels of factor 'Reef' within level '*Microcanthus joyceae*' of factor 'Species'

Comparison	ddf	t-value	P-value
**Tropical, Cold Temperate**	22	3.897	**<0.001**
**Tropical, Warm Temperate**	27	2.340	**0.022**
**Tropical, Subtropical**	27	3.507	**<0.001**
**Cold Temperate, Warm Temperate**	33	0.611	0.558
**Cold Temperate, Subtropical**	33	1.190	0.249
**Warm Temperate, Subtropical**	38	1.720	0.095

Bite rates in Australia

Table S10: ANOVA output of bite rates of warm-water and cool-water fish species in Australia and resulting pairwise tests. Note: **bold** P-values denote significant differences (P < 0.05). ndf = numerator degrees of freedom, SS = sum of squares, MS = mean square, ddf = denominator degrees of freedom.

Factor	ndf	SS	MS	ddf	F-value	P-value
**Reef**	3	0.230	0.077	124	5.758	**0.001**
**Species**	1	0.225	0.225	124	16.924	**<0.001**
**Reef×Species**	3	0.398	0.133	124	9.965	**<0.001**
**Residuals**	124	1.649	0.013			
**Total**	131	2.894				

Pairwise tests for levels of factor ‘Species’ within level ‘Tropical’ of factor ‘Reef’

Comparison	ddf	t-value	P-value
Abudefduf vaigiensis, Microcanthus joyceae	16	5.304	**<0.001**

Pairwise tests for levels of factor ‘Species’ within level ‘Subtropical’ of factor ‘Reef’

Comparison	ddf	t-value	P-value
Abudefduf vaigiensis, Microcanthus joyceae	33	3.242	**0.002**

Pairwise tests for levels of factor ‘Species’ within level ‘Warm Temperate’ of factor ‘Reef’

Comparison	ddf	t-value	P-value
Abudefduf vaigiensis, Microcanthus joyceae	41	4.444	**<0.001**

Pairwise tests for levels of factor ‘Species’ within level ‘Cold Temperate’ of factor ‘Reef’

Comparison	ddf	t-value	P-value
Abudefduf vaigiensis, Microcanthus joyceae	34	6.724	**<0.001**

Term ‘Species×Reef’ for pairs of levels of factor ‘Reef’ within level ‘*Abudefduf vaigiensis*’ of factor ‘Species’

Comparison	ddf	t-value	P-value
**Tropical, Cold Temperate**	28	8.412	**<0.001**
**Tropical, Warm Temperate**	30	7.458	**<0.001**
**Tropical, Subtropical**	22	2.478	**0.025**
**Cold Temperate, Warm Temperate**	42	0.126	0.903
**Cold Temperate, Subtropical**	34	3.999	**<0.001**
**Warm Temperate, Subtropical**	36	3.770	**0.001**

Term ‘Species×Reef’ for pairs of levels of factor ‘Reef’ within level ‘*Microcanthus joyceae*’ of factor ‘Species’

Comparison	ddf	t-value	P-value
**Tropical, Cold Temperate**	22	3.647	**0.001**
**Tropical, Warm Temperate**	27	2.274	**0.028**
**Tropical, Subtropical**	27	3.671	**0.001**
**Cold Temperate, Warm Temperate**	33	0.224	0.830
**Cold Temperate, Subtropical**	33	2.022	**0.049**
**Warm Temperate, Subtropical**	38	1.656	0.105

Prey attraction time in Australia

Table S11: ANOVA output of prey attraction time (sec) of warm-water and cool-water fish species in Australia and resulting pairwise tests. Note: **bold** P-values denote significant differences (P < 0.05). ndf = numerator degrees of freedom, SS = sum of squares, MS = mean square, ddf = denominator degrees of freedom.

Factor	ndf	SS	MS	ddf	F-value	P-value
**Reef**	3	21.109	7.036	124	5.534	**0.002**
**Species**	1	0.174	0.174	124	0.137	0.714
**Reef×Species**	3	18.553	6.185	124	4.864	**0.004**
**Residuals**	124	157.680	1.272			
**Total**	131	200.040				

**Pairwise tests for levels of factor 'Species' within level 'Tropical' of factor 'Reef'**			
**Comparison**	**ddf**	**t-value**	**P-value**
Abudefduf vaigiensis, Microcanthus joyceae	16	2.097	0.057
**Pairwise tests for levels of factor 'Species' within level 'Subtropical' of factor 'Reef'**			
**Comparison**	**ddf**	**t-value**	**P-value**
Abudefduf vaigiensis, Microcanthus joyceae	33	0.744	0.460
**Pairwise tests for levels of factor 'Species' within level 'Warm Temperate' of factor 'Reef'**			
**Comparison**	**ddf**	**t-value**	**P-value**
Abudefduf vaigiensis, Microcanthus joyceae	41	3.573	**<0.001**
**Pairwise tests for levels of factor 'Species' within level 'Cold Temperate' of factor 'Reef'**			
**Comparison**	**ddf**	**t-value**	**P-value**
Abudefduf vaigiensis, Microcanthus joyceae	34	3.674	**0.001**
**Term 'Species×Region" for pairs of levels of factor 'Reef' within level '*Abudefduf vaigiensis*' of factor 'Species'**			
**Comparison**	**ddf**	**t-value**	**P-value**
**Tropical, Cold Temperate**	28	2.783	**0.010**
**Tropical, Warm Temperate**	30	2.065	**0.046**
**Tropical, Subtropical**	22	1.150	0.263
**Cold Temperate, Warm Temperate**	42	0.065	0.951
**Cold Temperate, Subtropical**	34	5.277	**<0.001**
**Warm Temperate, Subtropical**	36	3.909	**<0.001**
**Term 'Species×Region' for pairs of levels of factor 'Reef' within level '*Microcanthus joyceae*' of factor 'Species'**			
**Comparison**	**ddf**	**t-value**	**P-value**
**Tropical, Cold Temperate**	22	2.695	**0.014**
**Tropical, Warm Temperate**	27	1.292	0.206
**Tropical, Subtropical**	27	2.182	**0.037**
**Cold Temperate, Warm Temperate**	33	1.702	0.106
**Cold Temperate, Subtropical**	33	0.067	0.950
**Warm Temperate, Subtropical**	38	1.487	0.142

Retreat rates in Australia

Table S12: ANOVA output of retreat rates of warm-water and cool-water fish species in Australia and resulting pairwise tests. Note: **bold** P-values denote significant differences (P < 0.05). ndf = numerator degrees of freedom, SS = sum of squares, MS = mean square, ddf = denominator degrees of freedom.

Factor	ndf	SS	MS	ddf	F-value	P-value
**Reef**	3	0.011	0.004	124	6.198	**<0.001**
**Species**	1	<0.001	<0.001	124	0.050	0.821
**Reef×Species**	3	0.004	0.001	124	2.148	0.088
**Residuals**	124	0.071	<0.001			
**Total**	131	0.087				

Pairwise tests for pairs of levels of fixed factor 'Reef'

Comparison	ddf	t-value	P-value
**Tropical, Cold Temperate**	50	1.738	0.0889
**Tropical, Warm Temperate**	57	1.715	0.0836
**Tropical, Subtropical**	49	2.577	**0.012**
**Cold Temperate, Warm Temperate**	75	0.480	0.6414
**Cold Temperate, Subtropical**	67	4.038	**<0.001**
**Warm Temperate, Subtropical**	74	3.692	**<0.001**

Escape rates in Australia

Table S13: ANOVA output of escape rates of warm-water and cool-water fish species in Australia and resulting pairwise tests. Note: **bold** P-values denote significant differences (P < 0.05). df = degrees of freedom, SS = sum of squares, MS = mean square.

Factor	ndf	SS	MS	ddf	F-value	P-value
**Reef**	3	0.002	<0.001	124	4.6483	**0.005**
**Species**	1	<0.001	<0.001	124	4.2028	**0.042**
**Reef×Species**	3	<0.001	<0.001	124	0.84555	0.463
**Res**	124	0.020	<0.001			
**Total**	131	0.023				

Pairwise tests for pairs of levels of fixed factor 'Reef'

Comparison	ddf	t-value	P-value
**Tropical, Cold Temperate**	50	2.842	**0.006**
**Tropical, Warm Temperate**	57	3.208	**0.001**
**Tropical, Subtropical**	49	2.095	**0.037**
**Cold Temperate, Warm Temperate**	75	1.277	0.214
**Cold Temperate, Subtropical**	67	0.989	0.329
**Warm Temperate, Subtropical**	74	2.145	0.034

Minimum distance to prey in Australia

Table S14: ANOVA output of minimum distance to prey (cm) of warm-water and cool-water fish species in Australia and resulting pairwise tests. Note: **bold** P-values denote significant differences (P < 0.05). ndf = numerator degrees of freedom, SS = sum of squares, MS = mean square, ddf = denominator degrees of freedom.

Factor	ndf	SS	MS	ddf	F-value	P-value
**Reef**	3	11.356	3.785	124	13.224	<0.001
**Species**	1	26.150	26.150	124	91.356	<0.001
**Reef×Species**	3	10.988	3.663	124	12.795	< 0.001
**Residuals**	124	35.494	0.286			
**Total**	131	102.09				

Pairwise tests for levels of factor ‘Species’ within level ‘Tropical’ of factor ‘Reef’

Comparison	ddf	t-value	P-value
Abudefduf vaigiensis, Microcanthus joyceae	16	1.174	0.268

Pairwise tests for levels of factor ‘Species’ within level ‘Subtropical’ of factor ‘Reef’

Comparison	ddf	t-value	P-value
Abudefduf vaigiensis, Microcanthus joyceae	33	7.774	**<0.001**

Pairwise tests for levels of factor ‘Species’ within level ‘Warm Temperate’ of factor ‘Reef’

Comparison	ddf	t-value	P-value
Abudefduf vaigiensis, Microcanthus joyceae	41	5.943	**<0.001**

Pairwise tests for levels of factor ‘Species’ within level ‘Cold Temperate’ of factor ‘Reef’

Comparison	ddf	t-value	P-value
Abudefduf vaigiensis, Microcanthus joyceae	34	10.304	**<0.001**

Term ‘Species×Reef’ for pairs of levels of factor ‘Reef’ within level ‘*Abudefduf vaigiensis*’ of factor ‘Species’

Comparison	ddf	t-value	P-value
**Tropical, Cold Temperate**	28	2.451	**0.022**
**Tropical, Warm Temperate**	30	2.309	**0.026**
**Tropical, Subtropical**	22	0.898	0.386
**Cold Temperate, Warm Temperate**	42	0.619	0.533
**Cold Temperate, Subtropical**	34	3.375	**0.001**
**Warm Temperate, Subtropical**	36	3.2447	**0.002**

Term ‘Species×Reef’ for pairs of levels of factor ‘Reef’ within level ‘*Microcanthus joyceae*’ of factor ‘Species’

Comparison	ddf	t-value	P-value
**Tropical, Cold Temperate**	22	6.725	**<0.001**
**Tropical, Warm Temperate**	27	3.500	**0.002**
**Tropical, Subtropical**	27	11.565	**<0.001**
**Cold Temperate, Warm Temperate**	33	1.429	0.155
**Cold Temperate, Subtropical**	33	1.3684	0.168
**Warm Temperate, Subtropical**	38	2.627	**0.012**

Chase rates in Australia

Table S15: ANOVA output of chase rates of warm-water and cool-water fish species in Australia and resulting pairwise tests. Note: **bold** P-values denote significant differences (P < 0.05). ndf = numerator degrees of freedom, SS = sum of squares, MS = mean square, ddf = denominator degrees of freedom.

Factor	ndf	SS	MS	ddf	F-value	P-value
**Reef**	3	<0.001	<0.001	124	2.284	0.070
**Species**	1	<0.001	<0.001	124	9.000	**0.003**
**Reef×Species**	3	<0.001	<0.001	124	1.555	0.200
**Residuals**	124	0.006	<0.001			
**Total**	131	0.007				

Fish Densities in Australia

Table S16: ANOVA output of densities of warm-water and cool-water fish species in regions in Australia and resulting pairwise tests. Note: **bold** P-values denote significant differences (P < 0.05). ndf = numerator degrees of freedom, SS = sum of squares, MS = mean square, ddf = denominator degrees of freedom.

Factor	ndf	SS	MS	ddf	F-value	P-value
**Reef**	3	7.022	2.341	204	4.693	**0.003**
**Species**	1	2.558	2.558	204	5.128	**0.024**
**Reef×Species**	3	2.045	0.682	204	1.367	0.256
**Residuals**	204	101.74	0.499			
**Total**	211	115.76				

**Pairwise comparisons of fixed factor 'Reef' for Australian fish densities**			
**Comparison**	**ddf**	**t value**	**P-value**
**Tropical, Subtropical**	66	4.027	**<0.001**
**Tropical, Warm Temperate**	116	2.766	**0.007**
**Tropical, Cold Temperate**	98	2.663	**0.009**
**Subtropical, Warm Temperate**	106	1.362	0.174
**Subtropical, Cold Temperate**	88	1.981	**0.049**
**Warm Temperate, Cold Temperate**	138	0.583	0.568

Minimum distance to prey in Japan

Table S17: ANOVA output of minimum distance to prey (cm) of warm-water and cool-water fish species in Japan and resulting pairwise tests. Note: **bold** P-values denote significant differences (P < 0.05). ndf = degrees of freedom, SS = sum of squares, MS = mean square, ddf = denominator degrees of freedom.

Factor	ndf	SS	MS	ddf	F-value	P value
**Species**	1	1.043	1.043	51	2.990	0.088
**Reef**	2	0.401	0.200	51	0.575	0.565
**Species×Reef**	2	6.151	3.076	51	8.819	**0.001**
**Residuals**	51	17.786	0.3488			
**Total**	56	29.187				

**Pairwise tests for levels of factor 'Species' within level 'Temperate' of factor 'Reef'**			
**Comparison**	**ddf**	**t-value**	**P-value**
Abudefduf vaigiensis, Microcanthus strigatus	28	5.464	**<0.001**
**Pairwise tests for levels of factor 'Species' within level 'Tropicalized of factor 'Reef'**			
**Comparison**	**ddf**	**t-value**	**P-value**
Abudefduf vaigiensis, Microcanthus strigatus	12	0.368	0.723
**Pairwise tests for levels of factor 'Species' within level 'Extreme' of factor 'Reef'**			
**Comparison**	**ddf**	**t-value**	**P-value**
Abudefduf vaigiensis, Microcanthus strigatus	11	0.930	0.367
**Term 'Species×Reef" for pairs of levels of factor 'Reef' within level '*Abudefduf vaigiensis*' of factor 'Species'**			
**Comparison**	**ddf**	**t-value**	**P-value**
**Temperate, Tropicalized**	19	1.478	0.155
**Temperate, Extreme**	22	2.700	**0.015**
**Tropicalized, Extreme**	13	0.866	0.407
**Term 'Species×Reef' for pairs of levels of factor 'Reef' within level '*Microcanthus strigatus*' of factor 'Species'**			
**Comparison**	**ddf**	**t-value**	**P-value**
**Temperate, Tropicalized**	21	2.739	**0.012**
**Temperate, Extreme**	17	2.407	**0.030**
**Tropicalized, Extreme**	10	0.614	0.557

Prey inspection rates in Japan

Table S18: ANOVA output of prey inspection rates of warm-water and cool-water fish species in Japan and resulting pairwise tests. Note: **bold** P-values denote significant differences (P < 0.05). ndf = numerator degrees of freedom, SS = sum of squares, MS = mean square, ddf = denominator degrees of freedom.

<table>
<tr><th>Factor</th><th>ndf</th><th>SS</th><th>MS</th><th>ddf</th><th>F-value</th><th>P value</th></tr>
<tr><td>Species</td><td>1</td><td>0.004</td><td>0.004</td><td>51</td><td>2.764</td><td>0.101</td></tr>
<tr><td>Reef</td><td>2</td><td>0.002</td><td>0.001</td><td>51</td><td>0.771</td><td>0.459</td></tr>
<tr><td>Species×Reef</td><td>2</td><td>0.030</td><td>0.015</td><td>51</td><td>11.149</td><td><0.001</td></tr>
<tr><td>Residuals</td><td>51</td><td>0.068</td><td>0.001</td><td></td><td></td><td></td></tr>
<tr><td>Total</td><td>56</td><td>0.102</td><td></td><td></td><td></td><td></td></tr>
<tr><td colspan="7">Pairwise tests for levels of factor ‘Species’ within level ‘Temperate’ of factor ‘Reef’</td></tr>
<tr><td colspan="2">Comparison</td><td>ddf</td><td colspan="2">t-value</td><td colspan="2">P-value</td></tr>
<tr><td colspan="2">Abudefduf vaigiensis, Microcanthus strigatus</td><td>28</td><td colspan="2">3.906</td><td colspan="2"><0.001</td></tr>
<tr><td colspan="7">Pairwise tests for levels of factor ‘Species’ within level ‘Tropicalized of factor ‘Reef’</td></tr>
<tr><td colspan="2">Comparison</td><td>ddf</td><td colspan="2">t-value</td><td colspan="2">P-value</td></tr>
<tr><td colspan="2">Abudefduf vaigiensis, Microcanthus strigatus</td><td>12</td><td colspan="2">1.825</td><td colspan="2">0.060</td></tr>
<tr><td colspan="7">Pairwise tests for levels of factor ‘Species’ within level ‘Extreme’ of factor ‘Reef’</td></tr>
<tr><td colspan="2">Comparison</td><td>ddf</td><td colspan="2">t-value</td><td colspan="2">P-value</td></tr>
<tr><td colspan="2">Abudefduf vaigiensis, Microcanthus strigatus</td><td>11</td><td colspan="2">2.293</td><td colspan="2">0.037</td></tr>
<tr><td colspan="7">Term ‘Species×Reef’ for pairs of levels of factor ‘Reef’ within level ‘Abudefduf vaigiensis’ of factor 'Species'</td></tr>
<tr><td colspan="2">Comparison</td><td>ddf</td><td colspan="2">t-value</td><td colspan="2">P-value</td></tr>
<tr><td colspan="2">Temperate, Tropicalized</td><td>19</td><td colspan="2">3.127</td><td colspan="2">0.005</td></tr>
<tr><td colspan="2">Temperate, Extreme</td><td>22</td><td colspan="2">4.548</td><td colspan="2"><0.001</td></tr>
<tr><td colspan="2">Tropicalized, Extreme</td><td>13</td><td colspan="2">0.133</td><td colspan="2">0.895</td></tr>
<tr><td colspan="7">Term ‘Species×Reef’ for pairs of levels of factor ‘Reef’ within level ‘Microcanthus strigatus’ of factor ‘Species’</td></tr>
<tr><td colspan="2">Comparison</td><td>ddf</td><td colspan="2">t-value</td><td colspan="2">P-value</td></tr>
<tr><td colspan="2">Temperate, Tropicalized</td><td>21</td><td colspan="2">2.096</td><td colspan="2">0.046</td></tr>
<tr><td colspan="2">Temperate, Extreme</td><td>17</td><td colspan="2">1.899</td><td colspan="2">0.075</td></tr>
<tr><td colspan="2">Tropicalized, Extreme</td><td>10</td><td colspan="2">0.865</td><td colspan="2">0.413</td></tr>
</table>

Bite rates in Japan

Table S19: ANOVA output of bite rates of warm-water and cool-water fish species in Japan and resulting pairwise tests. Note: **bold** P-values denote significant differences (P < 0.05). ndf = degrees of freedom, SS = sum of squares, MS = mean square, ddf = denominator degrees of freedom.

Factor	ndf	SS	MS	ddf	F-value	P value
**Species**	1	0.121	0.121	51	9.235	**0.004**
**Reef**	2	<0.001	<0.001	51	0.013	0.988
**Species×Reef**	2	0.431	0.216	51	16.524	**<0.001**
**Residuals**	51	0.666	0.013			
**Total**	56	1.136				

**Pairwise tests for levels of factor 'Species' within level 'Temperate' of factor 'Reef'**			
**Comparison**	**ddf**	**t-value**	**P-value**
Abudefduf vaigiensis, Microcanthus strigatus	28	5.875	**<0.001**
**Pairwise tests for levels of factor 'Species' within level 'Tropicalized of factor 'Reef'**			
**Comparison**	**ddf**	**t-value**	**P-value**
Abudefduf vaigiensis, Microcanthus strigatus	12	3.941	**<0.001**
**Pairwise tests for levels of factor 'Species' within level 'Extreme' of factor 'Reef'**			
**Comparison**	**ddf**	**t-value**	**P-value**
Abudefduf vaigiensis, Microcanthus strigatus	11	2.029	**0.049**
**Term 'Species×Reef' for pairs of levels of factor 'Reef' within level '*Abudefduf vaigiensis*' of factor 'Species'**			
**Comparison**	**ddf**	**t-value**	**P-value**
**Temperate, Tropicalized**	19	4.091	**<0.001**
**Temperate, Extreme**	22	3.063	**<0.001**
**Tropicalized, Extreme**	13	0.273	0.851
**Term 'Species×Reef' for pairs of levels of factor 'Reef' within level '*Microcanthus strigatus*' of factor 'Species'**			
**Comparison**	**ddf**	**t-value**	**P-value**
**Temperate, Tropicalized**	21	6.020	**<0.001**
**Temperate, Extreme**	17	4.649	**<0.001**
**Tropicalized, Extreme**	10	0.505	0.609

Chase rates in Japan

Table S20: ANOVA output of chase rates of warm-water and cool-water fish species in Japan and resulting pairwise tests. Note: **bold** P-values denote significant differences (P < 0.05). ndf = numerator degrees of freedom, SS = sum of squares, MS = mean square, ddf = denominator degrees of freedom.

Factor	ndf	SS	MS	ddf	F-value	P value
**Species**	1	<0.001	<0.001	51	0.291	0.601
**Reef**	2	<0.001	<0.001	51	1.023	0.369
**Species×Reef**	2	0.001	<0.001	51	5.858	**0.008**
**Residuals**	51	0.006	<0.001			
**Total**	56	0.007				

Pairwise tests for levels of factor ‘Species’ within level ‘Temperate’ of factor ‘Reef’

Comparison	ddf	t-value	P-value
Abudefduf vaigiensis, Microcanthus strigatus	28	3.643	**<0.001**

Pairwise tests for levels of factor ‘Species’ within level ‘Tropicalized of factor ‘Reef’

Comparison	ddf	t-value	P-value
Abudefduf vaigiensis, Microcanthus strigatus	12	1.166	0.308

Pairwise tests for levels of factor ‘Species’ within level ‘Extreme’ of factor ‘Reef’

Comparison	ddf	t-value	P-value
Abudefduf vaigiensis, Microcanthus strigatus	11	1.342	0.311

Term ‘Species×Reef’ for pairs of levels of factor ‘Reef’ within level ‘*Abudefduf vaigiensis*’ of factor ‘Species’

Comparison	ddf	t-value	P-value
**Temperate, Tropicalized**	19	2.133	**0.046**
**Temperate, Extreme**	22	1.777	0.089
**Tropicalized, Extreme**	13	0.966	0.355

Term ‘Species×Reef’ for pairs of levels of factor ‘Reef’ within level ‘*Microcanthus strigatus*’ of factor ‘Species’

Comparison	ddf	t-value	P-value
**Temperate, Tropicalized**	21	1.917	0.072
**Temperate, Extreme**	17	2.203	0.062
**Tropicalized, Extreme**	10	0.970	0.356

Fish Densities in Japan

Table S21: ANOVA output of densities of warm-water and cool-water fish species in reefs in Japan and resulting pairwise tests. Note: **bold** P-values denote significant differences ($P < 0.05$). ndf = numerator degrees of freedom, SS = sum of squares, MS = mean square, ddf = denominator degrees of freedom.

Factor	ndf	SS	MS	ddf	F-value	P -value
**Species**	1	0.565	0.565	26	0.032	0.862
**Reef**	2	73.452	36.726	26	2.082	0.141
**Reef×Species**	2	121.07	60.534	26	3.432	**0.040**
**Residuals**	26	458.670	17.641			
**Total**	31	653.22				

**Pairwise tests for levels of factor 'Species' within level 'Temperate' of factor 'Reef'**			
**Comparison**	**ddf**	**t-value**	**P-value**
Abudefduf vaigiensis, Microcanthus strigatus	8	1.921	0.089
**Pairwise tests for levels of factor 'Species' within level 'Tropicalized of factor 'Reef'**			
**Comparison**	**ddf**	**t-value**	**P-value**
Abudefduf vaigiensis, Microcanthus strigatus	10	1.154	0.272
**Pairwise tests for levels of factor 'Species' within level 'Extreme' of factor 'Reef'**			
**Comparison**	**ddf**	**t-value**	**P-value**
Abudefduf vaigiensis, Microcanthus strigatus	8	1.043	0.322
**Term 'Species×Reef" for pairs of levels of factor 'Reef' within level '*Abudefduf vaigiensis*' of factor 'Species'**			
**Comparison**	**ddf**	**t-value**	**P-value**
**Extreme, Tropicalized**	9	0.475	0.705
**Extreme, Temperate**	8	0.088	1.000
**Tropicalized, Temperate**	9	0.556	0.675
**Term 'Species×Reef' for pairs of levels of factor 'Reef' within level '*Microcanthus strigatus*' of factor 'Species'**			
**Comparison**	**ddf**	**t-value**	**P-value**
**Extreme, Tropicalized**	9	0.589	0.565
**Extreme, Temperate**	8	2.756	**0.023**
**Tropicalized, Temperate**	9	2.892	**0.018**

Prey attraction time in Japan

Table S22: ANOVA output of prey attraction time (sec) of warm-water and cool-water fish species in Japan and resulting pairwise tests. Note: **bold** P-values denote significant differences (P < 0.05). ndf = numerator degrees of freedom, SS = sum of squares, MS = mean square, ddf = denominator degrees of freedom.

Factor	ndf	SS	MS	ddf	F-value	P value
**Species**	1	3.669	3.669	51	3.302	0.072
**Reef**	2	13.466	6.733	51	6.061	**0.004**
**Species×Reef**	2	15.184	7.592	51	6.834	**0.004**
**Residuals**	51	56.661	1.111			
**Total**	56	86.517				

**Pairwise tests for levels of factor 'Species' within level 'Temperate' of factor 'Reef'**			
**Comparison**	**ddf**	**t-value**	**P-value**
Abudefduf vaigiensis, Microcanthus strigatus	28	2.415	**0.023**
**Pairwise tests for levels of factor 'Species' within level 'Tropicalized of factor 'Reef'**			
**Comparison**	**ddf**	**t-value**	**P-value**
Abudefduf vaigiensis, Microcanthus strigatus	12	2.615	**0.025**
**Pairwise tests for levels of factor 'Species' within level 'Extreme' of factor 'Reef'**			
**Comparison**	**ddf**	**t-value**	**P-value**
Abudefduf vaigiensis, Microcanthus strigatus	11	1.212	0.251
**Term 'Species×Reef" for pairs of levels of factor 'Reef' within level '*Abudefduf vaigiensis*' of factor 'Species'**			
**Comparison**	**ddf**	**t-value**	**P-value**
**Temperate, Tropicalized**	19	0.179	0.857
**Temperate, Extreme**	22	0.019	0.986
**Tropicalized, Extreme**	13	0.117	0.905
**Term 'Species×Reef' for pairs of levels of factor 'Reef' within level '*Microcanthus strigatus*' of factor 'Species'**			
**Comparison**	**ddf**	**t-value**	**P-value**
**Temperate, Tropicalized**	21	5.757	**0.001**
**Temperate, Extreme**	17	3.108	0.067
**Tropicalized, Extreme**	10	0.764	0.454

Retreat rates in Japan

Table S23: ANOVA output of retreat rates of warm-water and cool-water fish species in Japan and resulting pairwise tests. Note: **bold** P-values denote significant differences (P < 0.05). ndf = numerator degrees of freedom, SS = sum of squares, MS = mean square, ddf = denominator degrees of freedom.

Factor	ndf	SS	MS	ddf	F-value	P value
**Species**	1	<0.001	<0.001	51	0.046	0.837
**Reef**	2	0.003	0.001	51	1.344	0.273
**Species×Reef**	2	0.002	0.001	51	1.128	0.329
**Residuals**	51	0.053	0.001			
**Total**	56	0.058				

Escape rates in Japan

Table S24: ANOVA output of escape rates of warm-water and cool-water fish species in Japan and resulting pairwise tests. Note: **bold** P-values denote significant differences (P < 0.05). ndf = numerator degrees of freedom, SS = sum of squares, MS = mean square, ddf = denominator degrees of freedom.

Factor	ndf	SS	MS	ddf	F-value	P value
**Species**	1	<0.001	<0.001	51	0.270	0.612
**Reef**	2	<0.001	<0.001	51	0.394	0.683
**Species×Reef**	2	<0.001	<0.001	51	2.275	0.110
**Residuals**	51	0.004	<0.001			
**Total**	56	0.004				

Behavioural responses to marine heatwave at a temperate reef in Japan

Table S25: MANOVA output of foraging behaviours of warm-water and cool-water fish species at the temperate reef in Japan before and during the unprecedented marine heatwave and resulting pairwise tests. ndf = numerator degrees of freedom, ddf = denominator degrees of freedom, SS = sum of squares, MS = mean square. Bonferroni corrections were applied to the secondary MANOVA ($P < 0.025$).

Factor	ndf	SS	MS	ddf	F-value	P-value
**Species**	1	392.32	392.32	26	2.407	0.100
**Heatwave**	1	335.59	335.59	26	2.059	0.128
**Species×Heatwave**	1	360.4	360.4	26	2.211	0.117
**Residuals**	26	4237.6	4237.6			
**Total**	29	5294.8				
